# Automaticity of Early Sexual Attention: An Event-Related Potential Study

**DOI:** 10.1177/10790632211024241

**Published:** 2021-07-08

**Authors:** Anastasios Ziogas, Benedikt Habermeyer, Wolfram Kawohl, Elmar Habermeyer, Andreas Mokros

**Affiliations:** 1 University Hospital of Psychiatry Zurich, Switzerland; 2 Department of Psychiatry and Psychotherapy, Psychiatric Services Aargau, Brugg, Switzerland; 3 Department of Psychiatry, Psychotherapy and Psychosomatics, Psychiatric Hospital, University of Zurich, Zurich, Switzerland; 4 FernUniversität in Hagen, Germany

**Keywords:** sexual preference, brain potentials, forensic assessment, paraphilic sexual interests

## Abstract

A promising line of research on forensic assessment of paraphilic sexual interest focuses on behavioral measures of visual attention using sexual stimuli as distractors. The present study combined event-related potentials (ERPs) with behavioral measures to investigate whether detection of a hidden sexual preference can be improved with ERPs. Normal variants of sexual orientation were used for a proof-of-concept investigation. Accordingly, 40 heterosexual and 40 gay men participated in the study. Within each group, half of the participants were instructed to hide their sexual orientation. The results showed that a match between sexual orientation and stimulus delays responses and influences ERP before motor responses. Late ERP components showed higher potential in differentiating hidden sexual preferences than motor responses, thereby showing how ERPs can be used in combination with reaction time measures to potentially facilitate the detection of hidden sexual preferences.

Visual attention allows us to quickly focus on single objects of interest within a complex environment. This automatic and involuntary orientation is modulated by the perceptual properties of a stimulus such as shape or color or by its emotional value (for a review see Sennwald et al., 2016; Vuilleumier, 2005). The emotional value of a stimulus, however, is likely to be appraised very differently between individuals. Only as a result of this individual appraisal, a stimulus event obtains an emotional meaning that may influence emotional, physiological, or behavioral reactions. According to information processing models of sexual arousal, a visual sexual stimulus first needs to be appraised as a meaningful sexual stimulus by that person to be followed by a reactive phase, where attentional resources are further guided toward this stimulus and genital arousal is triggered (Janssen et al., 2000). For example, in laboratory experiments, sexual images presented subliminally led to faster identification of sexual images presented subsequently (Spiering et al., 2006). Neural correlates (thalamo-amygdala projection) known for their role in instinctive, unconscious, and quick responses (Ledoux, 1993) were also linked to an automatic connection between the appraisal of a stimulus and the mere perception of the stimulus (Spiering & Everaerd, 2007).

## Reaction Time Measures in Attentional Tasks

Attentional tasks can employ sexual content targeted at individual sexual interests. Studies have been conducted with normal variants of sexual orientation (i.e., gay and heterosexual orientation) as well as with paraphilic sexual interests involving samples where subjects may not be willing to disclose their sexual interests (e.g., pedophilic interest).

Based on the assumption that people look longer at sexually attracting images, participants can be asked to complete innocuous tasks on different pictures while the viewing time is recorded unobtrusively. Early studies on such viewing time measures were able to link viewing times to sexuality-related traits (Rosenzweig, 1942) or differentiate sexual orientations (Zamansky, 1956). Viewing time measures have also been used in forensic settings (e.g., Harris et al., 1996). Based on these findings, it was assumed that visual attention might not fully explain automaticity of the mechanisms involved (Imhoff et al., 2012). It was also noted that automatic early processes could be better studied in tasks demanding visual attention under time pressure. Such task demands are, for instance, incorporated in the choice reaction time task (Wright & Adams, 1994) and the dot-probe task (Koster et al., 2004).

In both tasks, selective visual attention to a neutral stimulus (dot) is demanded, while a sexual image tailored to individual sexual interest serves as a distractor. Typically, the participants are instructed to localize the dot as quickly as possible. If visual attention toward sexual content is guided through initial automatic processes, there should always be a delay when confronted with such distractors. Slower responses in the presence of a sexual stimulus have also been labeled as *sexual content-induced delay* (Geer & Bellard, 1996). The choice reaction time task displays both the sexual image and a randomly placed dot simultaneously while participants are asked to localize the dot as quickly as possible. This condition is typically compared to conditions with neutral images, and the difference in reaction times between these conditions captures this sexual content-induced delay.

The dot-probe task is a more variable task. Its simplest form requires the simultaneous presentation of two pictures followed by a dot at one of the picture positions. In the congruent condition, the dot follows the relevant sexual image, and in the incongruent condition, the dot follows the irrelevant neutral image. Another condition uses only neutral pictures and serves as a baseline (often conditions without a dot are also used). Longer reaction times have been reported when the dot was presented at the position of the sexual image (Prause et al., 2008). This was explained by the sexual picture grabbing visual attention and tying up processing so that the resources to process the subsequent dot are missing. In other studies, shorter reaction times have been assumed and observed when the dot is presented at the position of the sexual image (Kagerer et al., 2014; Snowden et al., 2016). It was hypothesized that visual attention is focused on the sexually preferred picture, and neutral stimuli presented in the same location afterward benefit from the already allocated visual attention. Using the dot-probe task, further inferences can be made on this facilitated processing of the dot and its relation to vigilance and difficulty in disengaging. Difficulty in disengaging from the sexual picture would result in a delayed response in incongruent trials compared to the neutral baseline. Vigilance would be reflected in quicker responses to congruent trials compared to the neutral baseline (Koster et al., 2004). These tasks were able to describe differences between groups with different sexual interests (e.g., Santtila et al., 2009; Snowden et al., 2016) and differentiate child sexual abusers from nonsexual offenders (e.g., Mokros et al., 2013).

These two tasks show numerous advantages over other tasks in the field. Like others, they also rely on performance indices that only allow for faking bad (malingering in cognitive performance) with the goal of replacing self-reports that entail more easily falsifiable answers. In contrast to other tasks, however, a combination of these two maintains a focus on speed or accuracy as well as the use of sexual imagery as distracters and still allows for more inferences on the mechanisms involved. Although correct responses are also recorded, they mostly serve to control for compliance as displayed in accuracy because a correct response itself does not pose much of a challenge in these two tasks. An overly high percentage of incorrect responses would therefore hint at noncompliance with task instructions. Reaction times, in contrast, are a more meaningful measure because they allow quantifying performance through speed. Delaying a response on purpose within a still plausible time range at the millisecond level would seem unlikely.

Disadvantages can be noted as well. Like all attentional tasks, these two also rely on behavioral motoric executions (e.g., press of a button) to measure reaction times. When differentiating sexual interests, the relevant effects on reaction times are usually visible at later stages (e.g., 900 ms into the reactions). At this stage, conscious motoric execution is already possible, and therefore conscious faking might also be possible (Gronau et al., 2005; Sip et al., 2013). Furthermore, strategies of mental dissociation can be employed to perform tasks without delays. Studies have rarely focused on participants instructed to bypass such attentional tasks with a focus on sexual preference. It is also problematic to infer mental mechanisms prior to motoric executions using only measurements from later processing stages (e.g., 900 ms and beyond).

## Electrophysiological Measures

Earlier cognitive processes can be investigated using event-related brain potentials (ERPs). Usually in ERP studies, electroencephalogram (EEG) is recorded during different experimental conditions, such as viewing 30 neutral and 30 sexual pictures presented in random order. Then, the EEG segments during the different conditions can be averaged in the form of an amplitude mean (µV) and related to an external event like the presentation of a picture (e.g., ERPs averaged over 30 neutral and ERPs averaged over 30 sexual pictures). The averages of different conditions can then be compared. This allows for measurement of the cognitive processes as early as the onset of a picture (0 ms) even before any motoric executions are possible.

Previous work on neuroelectric correlates has shown how ERPs are capable of differentiating sexual arousal from other emotional states (for a review, see Ziogas et al., 2020). This was shown in late brain potentials (Hietanen et al., 2014) as well as early ones (Alho et al., 2015; Briggs & Martin, 2009) and even subliminal ones (Legrand et al., 2013). Originally, early potentials (100–200 ms after stimulus onset) were assumed to be influenced more by physical properties of a stimulus and less prone to cognitive processes, whereas, later potentials (ca. 500–1,000 ms after onset) would show the reverse pattern (Donchin et al., 1978). ERP studies using erotic pictures often reported findings in early negative components (early posterior negativity [EPN] ca. 150–200 ms after onset) at posterior and occipital sites (Bailey et al., 2012; Legrand et al., 2013; Prause, Steele, Staley, Sabatinelli, & Hajcak, 2015; Schupp et al., 2004). This EPN has also been linked to penile erection (Ponseti et al., 2009). A later, positive slow wave (PSW; 250–500 ms) was also frequently reported in studies on erotic pictures (Briggs & Martin, 2009; Deweese et al., 2016; Feng, Wang, Wang, et al., 2012; Hietanen et al., 2014; Oliver et al., 2016; Price et al., 2012). Although there is substantial work on visual sexual stimuli and ERPs, only a few studies have looked at paraphilic sexual interests or the use of ERPs in differentiating people exhibiting paraphilic sexual interest from healthy subjects (Howard et al., 1994; Knott et al., 2016; Waismann et al., 2003).

ERP studies aiming to segregate sexual preferences usually employed different sexual images matching the sexual preferences of the participating groups. Participants were usually asked to view the pictures passively or to rate them with regard to their emotional valence. Healthy subjects and subjects with paraphilic sexual interests often showed more pronounced ERPs to pictures matching their preferences than those not matching their preferences. For example, higher late amplitude measures were recorded in paraphilic individuals compared to controls when paraphilic pictures were shown (Waismann et al., 2003). Such ERPs were also correlated with self-reports on sexual preferences in both regular and paraphilic sexual interests (Knott et al., 2016; Waismann et al., 2003). While such results are based on averaged ERPs in very early time windows (e.g., 200–450 ms), no studies have tested whether subjects can also suppress such early sexual responses by utilizing similar strategies of mental dissociation to, say, trick penile plethysmography or measures of attentional tasks. In addition, the ERP studies required only passive viewing of the sexual pictures without demanding visual attention under time pressure. Thus, although these studies measured ERPs in early time windows before motoric executions are possible, they are not adequately designed to differentiate the aspect of automaticity as assumed in the attentional tasks described above. In contrast, studies recording early ERPs during attentional tasks under time pressure did not use such measures to differentiate between sexual preferences (e.g., Feng, Wang, Liu, et al., 2012). More problems can be found in the preparation of the sexual pictures used in such ERP studies.

Generally, when sexual images were used in ERP studies, only on rare occasions were the images controlled for other emotional dimensions, such as emotional arousal and emotional valence (Kuhr et al., 2013; van Lankveld & Smulders, 2008). Physical image properties such as luminance and complexity can influence visual perception as well as ERPs and should be controlled (Willenbockel et al., 2010). None of the studies have so far used ERPs to examine early neuroelectric correlates of sexual preference while simultaneously controlling for physical properties and other emotional dimensions in speed measures of attention. In addition to image properties, participant characteristics can further influence sexual responses to those images. Studies have shown that measured sexual desire (Prause et al., 2008), sexual inhibition, and sexual excitation (Janssen et al., 2002b) are associated with attentional and physiological responses to sexual imagery.

## The Present Study

In the present study, the choice reaction time task and the dot-probe task were used in combination with ERP recordings. Different stimulus sets were used so that effects could be attributed mostly to sexual content, while emotional arousal, valence, and physical stimulus properties were kept constant. The reaction time measures in the choice reaction time task were used to replicate sexual content-induced delay during simultaneous presentation of a sexual picture and a dot. Reaction time measures from the dot-probe task were used to investigate whether visual attention allocated to a sexual picture captures the resources needed for the processing of a dot presented subsequently at the same location. This resulted in longer reaction times when the dot was presented in the same position as the sexual picture. Faster responses could mean that attention allocated at the location of the sexual picture facilitates the processing of the subsequent dot. Results from the dot-probe task can further be used to characterize this facilitated processing with mechanisms of vigilance or difficulty in disengaging from the sexual picture.

An advantage of using two tasks was that only slight modifications were allowed for minimal eye movements during EEG recordings without influencing the performance measures. In this way, ERP prior to reaction time measures was used to infer initial mechanisms at the early processing stage of the EPN or the PSW. To investigate the degree of automaticity involved in all the processing stages and how participants could possibly counteract this automaticity and hide their sexual preference, a further experimental manipulation was introduced.

Two groups of heterosexual and gay men were recruited and instructed to lie about their sexual preferences and act as if they had opposite sexual preferences or to be truthful throughout the entire experiment. Furthermore, characteristics such as sexual desire, inhibition, and excitation were compared across groups.

Given the similar results in reaction time (e.g., Santtila et al., 2009) and ERP measures (e.g., Howard et al., 1994) for both heterosexual and gay samples, no general differences between heterosexual and gay participants were expected in the present study. Although the cognitive costs of lying can be described with reaction time measures or ERPs (Suchotzki et al., 2017), if processing of sexually relevant pictures is really guided by automatic processes, deceptive attempts should fail, and no differences should be observed between the lying and truthful groups in the present study.

Automatic processing of sexual pictures that match sexual preference (in terms of gender) should lead to sexual content-induced delays in the reaction times (e.g., Santtila et al., 2009; Snowden et al., 2016) and more pronounced ERPs (more negative EPN and more positive PSW) for all participants (e.g., Costell et al., 1972; Hietanen et al., 2014; Howard et al., 1992). When sexual pictures are shown in explicit versions (e.g., nude instead of clothed), this usually leads to longer reaction times (Santtila et al., 2009) and more pronounced ERPs (Prause, Steele, Staley, & Sabatinelli, 2015) but only when the sexual pictures match individual gender preferences (e.g., Legrand et al., 2013). If the present study could replicate these results, the choice reaction time task or the dot-probe task might further clarify whether automatic processing of sexual pictures leads to induced delays through mechanisms of vigilance or difficulty in disengaging. Overall, the hypotheses of the present study were as follows (this study was preregistered at https://aspredicted.org/ and the .pdf is available from https://aspredicted.org/zj2y2.pdf):

**Hypothesis 1:** Participants were expected to have longer reaction times and more pronounced ERPs for pictures matching their sexual preference.
**Hypothesis 2:** Participants were expected to have longer reaction times and more pronounced ERPs for naked pictures if the picture gender matched with participants’ sexual preference.
**Hypothesis 3:** When participants lied about their sexual preference, no effects were expected to be observed on reaction times or ERPs; therefore, no differences were expected between lying and truthful groups.

## Methods

We have reported, in this study, how we determined the sample size, all data exclusions (if any), all manipulations, and all measures taken.

### Participants

Gay and heterosexual men were invited through emails and leaflets to participate in this study. All participants had normal or corrected-to-normal vision. The sample of 80 participants (mean age = 24.75 years, *SD* = 4.95) were randomly divided into one of two study groups, a truthful or lying condition, by sexual orientation, with 20 men in each group (e.g., 20 gay men in the lying gay group were instructed to mimic a heterosexual orientation during the study).

The study was approved by the local ethics committee (Ethics Committee Zurich), and every participant signed an informed consent form. A power analysis using G*Power (Faul et al., 2009) indicated that 20 participants in each group would be sufficient to achieve 95% statistical power in a two-factor mixed design with a large effect size at the conventional Type I error rate of 5%.

### Stimuli

The main study required clothed and naked picture sets that could be differentiated based on the criterion of sexual attractiveness in relation to participants’ sexual orientation. Pictures were selected in a pilot study involving 20 gay men and 20 heterosexual men who did not take part in the main study, rating 480 different images with 120 pictures for each of four categories (male and female, clothed, and naked). The pictures were similar in terms of background (uniform white), ethnicity, and postures of the models depicted.

The pilot study participants rated all the pictures on three emotional dimensions—valence, arousal, and sexual attractiveness on a Likert-type scale ranging from 1 (*lowest rating*) to 5 (*highest rating*). These ratings were used to ensure that the clad and nude picture categories differed in sexual attractiveness, with valence and arousal controlled for (see Supplementary Material for more details). The final picture set included 60 clothed females, 60 naked females, 60 clothed males, and 60 naked males. These four sets of pictures were adjusted for luminance and complexity using the SHINE toolbox (Willenbockel et al., 2010).

### Apparatus

EEG was recorded with 32 electrodes referenced to FCz (BrainCap-MR 32 standard, 32 channels, Easycap). Scalp electrodes were placed in accordance with the international 10 to 20 system (Jasper, 1958), and impedances were kept below 20 kΩ. Data were collected at a sampling rate of 2,500 Hz (BrainAmp amplifier, Brain Products), and participants were seated approximately 1 m away from the screen (Dell S2209W 22" HD monitor with a resolution of 1920 × 1080). The pictures were shown at 8° (dot-probe task) and 11° (choice reaction time task) of the visual angle.

### Measures

#### Oldfield’s Edinburgh Inventory of Handedness

This questionnaire (Oldfield, 1971) uses 10 items to assess handedness. Each item consists of an activity (e.g., writing) that can be rated on a 5-point Likert-type scale ranging from -2 (left) to 2 (right). Using the laterality quotient LQ = (R − L)/(R + L) × 100, every participant was categorized as left-handed (LQ < 0) or right-handed (LQ > 0). This questionnaire was used to control for differences in handedness between the four groups.

#### Klein Sexual Orientation Grid

Within the random truthful and lying groups, the Klein Sexual Orientation Grid (KSOG; Klein et al., 1985) was used to check for self-reported sexual preference. This scale uses seven items on sexual orientation (i.e., sexual attraction, sexual behavior, sexual fantasies, emotional preference, social preference, self-identification, and heterosexual/gay lifestyle outcomes) that are addressed with responses on three dimensions (past, present, and future). Responses are given on a 7-point Likert-type scale ranging from 1 (*other sex only/heterosexual only*) to 7 (*same-sex only/gay only*). Cronbach’s alpha of .96 and a significant correlation with self-reported sexual orientation (*r* = .71) have been reported for the KSOG (Qualls et al., 2018). Average scores across the present time dimension were calculated. Values of 2.9 or less refer to heterosexual orientation, and values of 5.0 or above refer to gay orientation (Moore & Norris, 2005). All participants in the gay condition (truthful gay group, lying gay group) showed values of ≥ 5.0, and all participants in the heterosexual condition (truthful heterosexual group and lying heterosexual group) showed values of 2.9 or less.

#### Sexual Desire Inventory

The German-language short version of the Sexual Desire Inventory (SDI-2; Kuhn et al., 2014, based on Spector et al., 1996) was used to assess sexual desire. This questionnaire comprises 10 items from which scores for two different subscales can be calculated. The two subscales of the SDI-2 measure sexual desire for interaction (SDI-2 Scale 1) and sexual desire without interaction (SDI-2 Scale 2). Both scores were used to control for differences in sexual desire between the four groups.

#### Sexual Inhibition and Sexual Excitation

The German-language short form of the Sexual Inhibition and Sexual Excitation Scales (SIS/SES; de Albuquerque, 2012, based on Janssen et al., 2002a) was used to assess sexual inhibition and excitation. The SIS/SES consists of three subscales (SIS 1: sexual inhibition due to threat of performance failure; SIS 2: sexual inhibition due to threat of performance consequences; SES: sexual excitation) based on 14 four-point Likert-type items. The three scores were used to control for differences in sexual inhibition and excitation between the four groups.

#### Attentional network task

The attentional network task (ANT; Wang et al., 2015) was used to examine basic attentional performance without any emotional content. This task was programmed with Inquisit 5 Lab (Inquisit 5, Millisecond Software) and used to control for differences in attentional performance between the four groups. The ANT measures performance with reaction time difference scores on three attentional performance indices (ANT A: alerting, ANT O: orienting, and ANT E: executive control). These three outcome measures were calculated for each of the four groups.

#### Rating

The final picture set from the preliminary study (60 clothed females, 60 naked females, 60 clothed males, and 60 naked male pictures) was rated again in the main study (see Supplementary Material for more details). All pictures were rated on three emotional dimensions—valence, arousal, and sexual attractiveness—using a Likert-type scale ranging from 1 to 5 with 1 as the lowest and 5 as the highest rating in each scale (the task was programmed with Presentation [Neurobehavioral Systems, Albany, NY]). This resulted in three rating measures (valence, arousal, and sexual attractiveness) for both clothed and naked picture categories. These ratings were used to ensure that the clothed and naked picture categories selected in the preliminary study mostly differed in sexual attractiveness (while valence and arousal were controlled for) when rated again by the sample from the main study.

#### Choice reaction time task

**Figure 1.**
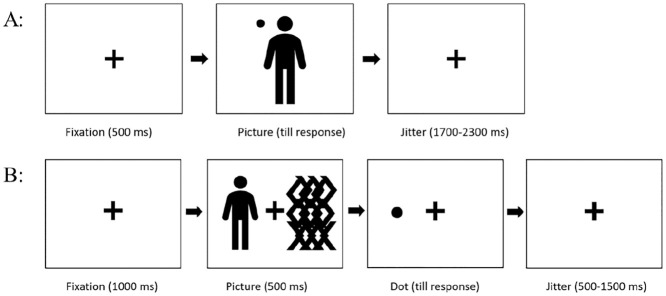
Study paradigm. (A) Example of a trial in the choice reaction time task. An example trial presents a picture with a dot in the upper left corner. (B) Example of a trial in the dot-probe task. The sequence shows a picture on the left and a neutral, scrambled picture on the right followed by a dot on the left, resulting in a congruent trial. Both figures do not accurately reflect the real relations in size.

The four picture categories were embedded in the choice reaction time task (all tasks except for the ANT were programmed with Presentation version 20.0 [Neurobehavioral Systems, Albany, NY]). Within the choice reaction time task, all selected pictures were presented randomly, resulting in 240 trials (60 trials per picture category). The entire task was split into four blocks with an equal number of trials. Each trial started with a blank screen (500 ms) and was followed by the simultaneous presentation of one picture (clothed female, naked female, clothed male, or naked male) at the center of the screen and a black dot (0.5 cm in diameter) randomly placed around the picture or at the center (9 possible positions). Participants were instructed to localize the dot accurately and as fast as possible using nine buttons of a keypad corresponding to the possible dot positions. The picture and the dot remained on the screen until a button was pressed. After the button was pressed, a blank screen (random jitter between 1,700 and 2,300 ms) followed, and the next trial was initiated. Reaction time was measured from the onset of the picture until the first button press. Answers were either correct or incorrect. Figure 1A depicts a typical trial sequence in the upper panel.

The choice reaction time task resulted in accuracy measures (percentage ratings for correct responses), reaction time measures, and EPN and PSW recordings for each of the four picture conditions.

#### Dot-probe task

The four picture categories were embedded in the dot-probe task. Figure 1B depicts a typical trial sequence used in this study. After a fixation cross (1,000 ms), the actual stimuli (left) paired with a scrambled picture (right) were displayed for 500 ms. This was followed by a dot on the left-hand side. The dot stayed on the screen until a response was provided. After a randomly jittered interval (500–1,500 ms), the next trial was initiated. The dot-probe task used in the present study comprised a total of 600 trials. Trials showing neutral scrambled pictures on both sides were used as a baseline (40 trials with a dot following on the right side, 40 trials with a dot following on the left side, and 40 trials without a dot). Only trials with a dot were used for data analysis. Apart from the 120 baseline trials, 480 trials showed one of the pictures either on the left-hand side (240 trials) or on the right-hand side (240 trials) with a neutral scrambled picture on the other side. Again, the dot could appear on the left side (160 trials), on the right side (160 trials), or not at all (160 trials, in the latter case, participants were instructed not to press any button and the trial continued automatically after 1,000 ms). When the dot was presented where a picture had previously been shown, the trial has been referred to as a *congruent* trial. When the dot was presented where a neutral scrambled picture had been shown, it is referred to as an *incongruent* trial. As congruent and incongruent trials have to be further differentiated as an experimental condition within the dot-probe task, more trials are needed to achieve an adequate signal-to-noise ratio for the ERPs (see below). The 360 picture trials followed by a dot consisted of 40 congruent and 40 incongruent trials for each of the four picture categories. Every single picture was displayed 2 times within the 600 trials of the dot-probe task. The task was split into six blocks with 100 trials each (see Supplementary Material for more details).

The dot-probe task resulted in accuracy measures (percentage ratings for correct responses) and reaction time measures for two different versions (congruent and incongruent) of each of the four picture conditions. EPN and PSW were recorded for each of the four picture conditions, irrespective of dot congruency.

### Procedure

Participants arrived one at a time and sat in a chair. They filled out questionnaires on handedness (Oldfield, 1971) and sexuality (KSOG, SDI-2, and SIS/SES), while EEG electrodes were placed on their heads. The instructions on lying followed (if applicable). The participants in the lying condition received a written explanation that they should act according to the other sexual orientation throughout the rest of the session, including all the experimental demands, without being informed about any of the effects of lying on reaction time measures. Participants within the lying condition confirmed their understanding of this instruction by writing down their true sexual orientation as well as the one they were lying about for the study. In addition, to familiarize themselves with the instruction on lying, participants filled out the KSOG a second time but this time according to the sexual orientation that they pretended to have for the rest of the study (see Supplementary Material for more details). The researcher stayed close to the participants during this initial briefing to answer potential questions about the instructions and to ensure proper comprehension before the actual experiment began. Male and female researchers were present throughout most sessions.

An experimental session always included the choice reaction time task, the dot-probe task, and the rating. The order of these three tasks was balanced within each group. During the short breaks between and within the tasks, participants in the lying condition were reminded about the instruction to lie during the experiment. At the end of the session, the ANT (Wang et al., 2015) was used to examine basic attentional performance without any emotional content. No EEG was recorded during the ANT, and participants within the lying condition were relieved from lying.

To minimize eye movement, a fixation cross was displayed in the center of the screen throughout all three tasks. Participants were instructed to focus on the cross while performing each task. For each task, initial practice trials were used to familiarize participants with the exercise. Practice trials always included pictures that were not used in the real tasks.

### Data Analysis

#### Behavioral data

Trials with reaction times that were 3 *SD* units below or above the group mean were excluded from further analysis in all tasks. Furthermore, one entire data set was excluded from analysis in the choice reaction time task (participant only answered correctly in 50% of the trials), and two entire data sets were excluded from analysis in the dot-probe task (48% and 49% correct trials overall, respectively).

The open-source software *R* (versions 3.2.4–3.6.1) and the “ez” package (Lawrence & Lawrence, 2016) were used for all statistical analyses in this study (both the preliminary study and the main study). An overall mixed-design ANOVA with the factors lying (2: lying vs. truthful), sexual orientation (2: heterosexual vs. gay), sexual match (2: match [picture category matches sexual orientation] vs. mismatch [picture category does not match sexual orientation]), and sexual explicitness (2: naked [sexually explicit] vs. clothed [sexually non-explicit]) was calculated for both accuracy (percentages) and reaction times. In the dot-probe task, congruency of the dot position was further added as a within-subject factor.

There were no statistically significant effects on any percentage of correct responses in either task. As hypothesized, only the reaction time results are discussed further below. No main effect of sexual orientation on reaction times was expected because no differences between heterosexual and gay men were assumed. The main effect of sexual match on reaction times with longer reaction times for matching pictures would represent support for Hypothesis 1. According to Hypothesis 2, longer reaction times were expected for naked pictures if the picture’s gender matched the participants’ sexual preferences, while no difference was expected between the naked and the clothed picture categories when pictures did not match sexual interest within each group. This would be supported by an interaction between the factors sexual match and explicitness of reaction times. According to Hypothesis 3, no differences were expected between the lying and the truthful groups. Therefore, no main effect of lying was expected on the reaction times.

In the rating task, the scores of the participants on the pictures of the gender matching sexual preference were used; and MANOVA, with picture clothing as the independent variable (clothed vs. naked) and the three ratings as the dependent variables, was calculated. The main effect of explicitness was expected with ratings on sexual attractiveness, distinguishing best between clothed and naked pictures.

#### ERP data

EEG data from the choice reaction time task and the dot-probe task were handled separately with the EEGLab toolbox available for MATLAB. The data were filtered with a high-pass filter of 0.5 Hz and a low-pass filter of 30 Hz cutoff. Independent component analysis was used (Bell & Sejnowski, 1995) to decompose the data into statistically independent components. The EEGLab plugin MARA (Winkler et al., 2011) was then used to automatically classify components representing various kinds of artifacts and remove them.

The continuous EEG segments were then segmented into stimulus-locked epochs based on the experimental task conditions defined by the different picture categories embedded in the tasks and baseline corrected (100 ms to stimulus onset). In the choice reaction time task and the dot-probe task, single trials were segmented with 200 ms prestimulus and 500 ms poststimulus intervals because signals before the first motoric responses were useful for the ERP analysis. Single epochs were excluded from further analysis if data points within the epochs exceeded a threshold of ± 80 µV. Within the choice reaction time task, grand means were calculated for the four picture conditions (clothed female, naked female, clothed male, and naked male pictures). Within the dot-probe task, the same four grand means were calculated (clothed female, naked female, clothed male, and naked male pictures). As the distinction between congruent and incongruent trials occurs at the stage when the dot is presented (500 ms after stimulus onset), this factor was omitted from the ERP data. As the focus was on EPN, activity recorded at the five most posterior and occipital electrodes (Pz, O1, O2, P3, and P4) was averaged, and the mean amplitude values (150–200 ms) were calculated.

These picture conditions were recategorized according to factors of the sexual match and sexual explicitness. An overall mixed-design ANOVA with the factors lying (2: lying vs. truthful), sexual orientation (2: heterosexual vs. gay), sexual match (2: match [picture category matches sexual orientation] vs. mismatch [picture category does not match sexual orientation]), and sexual explicitness (2: naked [sexually explicit] vs. clothed [sexually non-explicit]) was calculated for the EPN and PSW data.^1^ The same three data sets showing a high rate of incorrect responses were excluded.

No main effect of sexual orientation on ERPs was expected because no differences between heterosexual and gay men were assumed. A main effect of sexual match on ERPs (EPN [more negative] and PSW [more positive]) would support Hypothesis 1. According to Hypothesis 2, more pronounced ERPs were expected for naked pictures if the picture’s gender matched the participants’ sexual preferences, while no difference was expected between the naked and the clothed picture categories when pictures did not match sexual interest within each group. This would be supported by an interaction between the factors sexual match and explicitness of ERPs. According to Hypothesis 3, no differences were expected between the lying and truthful groups. Therefore, no main effect of lying was expected on the ERPs.

## Results

### Group Characteristics

Prior to testing the hypotheses, participant groups were compared in terms of potentially confounding variables (i.e., handedness, sexual desire, sexual excitation/inhibition, and general attentional performance). Table 1 provides an overview of the group characteristics. There were no group effects on age (in years) or handedness.^2^ There was an effect of group on the second scale of the SDI-2, *F*(3, 76) = 4.94, *p* = .003, η^2^ = 0.16, with the lying heterosexual group showing a significantly lower mean score than both the truthful and the lying gay groups (both *p* < .05, Tukey’s post hoc test). However, when both truthful groups (truthful heterosexual group and truthful gay group) were compared to the two lying groups (lying heterosexual group and lying gay group), there were no significant differences on this scale between the two (*p* > .05, independent samples *t*-test). There was no group effect on any of the SES/SIS subscales. There were also no group effects on any of the ANT performance measures.

**Table 1.** Group Characteristics.

Variables	HEA	HEF	GA	GF	Test statistic	*df*	*p* value
*N*	20	20	20	20			
Age (Mean, *SD*)	25.5 (6.2)	22.95 (3.4)	25.9 (4.3)	24.55 (5.1)	*F* = 1.43	3, 76	>.05
Age (Range)	20–42	20–34	21–39	19–43			
KSOG-T (Mean, *SD*)	1.93 (0.41)	1.91 (0.39)	5.71 (0.47)	5.78 (0.34)	*F* = 600.67	3, 76	<.01
KSOG-T (Range)	1.14–2.57	1.29–2.86	5.00–6.43	5.14–6.29			
KSOG-F (Mean, *SD*)		5.63 (0.65)		2.06 (0.36)	*t* = 21.29	38	<.01
KSOG-F (Range)		3.71–6.71		1.57–3.00			
Handedness (Right/Left)	19, 1	17, 3	18, 2	18, 2	χ^2^ = 1.11	49	>.05
SDI-2 Scale 1 (Mean, *SD*)	25.6 (5.0)	25.6 (4.6)	25.8 (4.4)	26.2 (2.4)	*F* = 0.10	3, 76	>.05
SDI-2 Scale 2 (Mean, *SD*)	24.0 (5.9)	20.9 (6.7)	25.8 (4.7)	27.7 (5.9)	*F* = 4.94	3, 76	<.05
SIS 1 (Mean, *SD*)	8.4 (2.2)	8.5 (2.1)	8.4 (1.4)	8.4 (1.4)	*F* = 0.01	3, 76	>.05
SIS 2 (Mean, *SD*)	10.5 (1.8)	9.9 (2.4)	9.4 (2.1)	9.6 (2.6)	*F* = 0.93	3, 76	>.05
SES (Mean, *SD*)	17.2 (2.5)	16.2 (2.5)	17.8 (2.3)	16.8 (2.2)	*F* = 1.62	3, 76	>.05
ANT A (Mean, *SD*)	−0.1 (0.1)	−0.1 (0.1)	−0.1 (0.1)	−0.1 (0.1)	*F* = 0.74	3, 76	>.05
ANT O (Mean, *SD*)	0.0 (0.1)	0.0 (0.1)	0.0 (0.1)	0.0 (0.1)	*F* = 1.45	3, 76	>.05
ANT E (Mean, *SD*)	0.1 (0.1)	0.1 (0.1)	0.1 (0.1)	0.1 (0.1)	*F* = 0.39	3, 76	>.05

*Note.* HEA = truthful heterosexual group, HEF = lying heterosexual group; GA = truthful gay group; GF = lying gay group; SD = standard deviation; KSOG-T = truthful scores from the Klein Sexual Orientation Grid; KSOG-F = fake scores from the Klein Sexual Orientation Grid (Klein et al., 1985); SDI-2 Scale 1 = sexual desire with interaction; SDI-2 Scale 2 = sexual desire without interaction (Kuhn et al., 2014 based on Spector et al., 1996); SIS 1 = sexual inhibition due to threat of performance failure; SIS 2 = sexual inhibition due to threat of performance consequences; SES = sexual excitation scale (de Albuquerque, 2012 based on Janssen et al., 2002a); ANT A = alerting from the attentional network task; ANT O = orienting from the attentional network task; ANT E = executive control from the attentional network task (Wang et al., 2015).

### Rating

The results demonstrated the comparability of the stimuli on valence and arousal, replicating the findings from the preliminary study (see Supplementary Material for more details).

### Choice Reaction Time Task

#### Accuracy

There were no significant main effects on the accuracy data, sexual match: *F*(1, 75) = 2.48, *p* = .120, η_p_^2^ < 0.01; sexual explicitness: *F*(1, 75) = 0.128, *p* = .720, η_p_^2^ < 0.01; and lying: *F*(1, 75) = 0.227, *p* = .635, η_p_^2^ < 0.01. There was no interaction between sexual match and lying, *F*(1, 75) = 1.46, *p* = .230, η_p_^2^ = 0.02, and between sexual match and explicitness, *F*(1, 75) = 1.91, *p* = .171, η_p_^2^ = 0.03. This supported the notion that participants displayed an equal amount of correct responses throughout all experimental conditions of the choice reaction time task.

#### Reaction times

**Figure 2.**
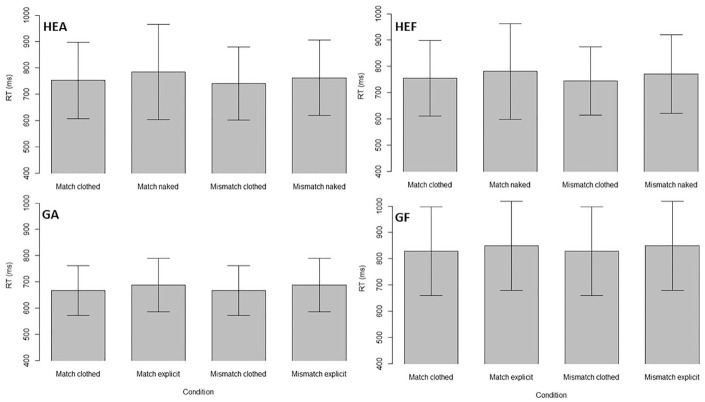
Mean reaction times from the choice reaction time task. *Note.* Mean reaction times (RT) and *SD* for all four groups and picture conditions (FC = clothed female, FN = naked female, MC = clothed male, MN = naked male, GA = truthful gay group, GF = lying gay group, HEA = truthful heterosexual group, and HEF = lying heterosexual group). RT = reaction times; SD = standard deviation.

Figure 2 shows the reaction time results from the choice reaction time task. There was a main effect of the sexual match on pictures reflecting one’s sexual preference, resulting in longer reaction times, *F*(1, 75) = 13.36, *p* < .001, η_p_^2^ = 0.15. Explicitness had an effect on reaction times with naked pictures resulting in longer reaction times than clothed pictures, *F*(1, 75) = 55.32, *p* < .001, η_p_^2^ = 0.42. In line with Hypothesis 1, there was an interaction effect of the sexual match on pictures reflecting one’s sexual preference, resutling in longer reaction times, *F*(1, 75) = 11.61, *p* = .001, η_p_^2^ = 0.13. The effect of sexual explicitness was more pronounced when pictures matched sexual preference. In addition, as expected, sexual orientation showed no main effect on reaction times, *F*(1, 75) = 0.21, *p* = .643, η_p_^2^ < 0.01. These results support Hypotheses 1 and 2. When the gender on the pictures matched the sexual preference of the participants, it resulted in a delayed response. In addition, when the gender on the pictures matched sexual preference, the naked pictures led to a stronger delay in responses compared to pictures that did not match sexual preference.

However, contrary to the expectations, lying also showed a main effect, with lying groups showing generally longer reaction times compared to the truthful groups, *F*(1, 75) = 4.39, *p* = .039, η_p_^2^ = 0.06. This contradicted the third hypothesis and showed that attempts at lying about one’s sexual preference increased reaction times in the choice reaction time task. There was also an interaction between lying and sexual match, showing that the delayed response in matching pictures was more pronounced in the truthful than the lying groups, *F*(1, 75) = 5.09, *p* = .026, η_p_^2^ = 0.06. Lying also interacted with sexual orientation, showing that the delay in untrue responses was higher in gay men than in heterosexual men, *F*(1, 75) = 4.10, *p* = .046, η_p_^2^ = 0.051. There was also a second-order interaction among sexual orientation, matching, and explicitness, *F*(1, 75) = 8.04, *p* = .005, η_p_^2^ = 0.01.

#### Early posterior negativity

**Figure 3.**
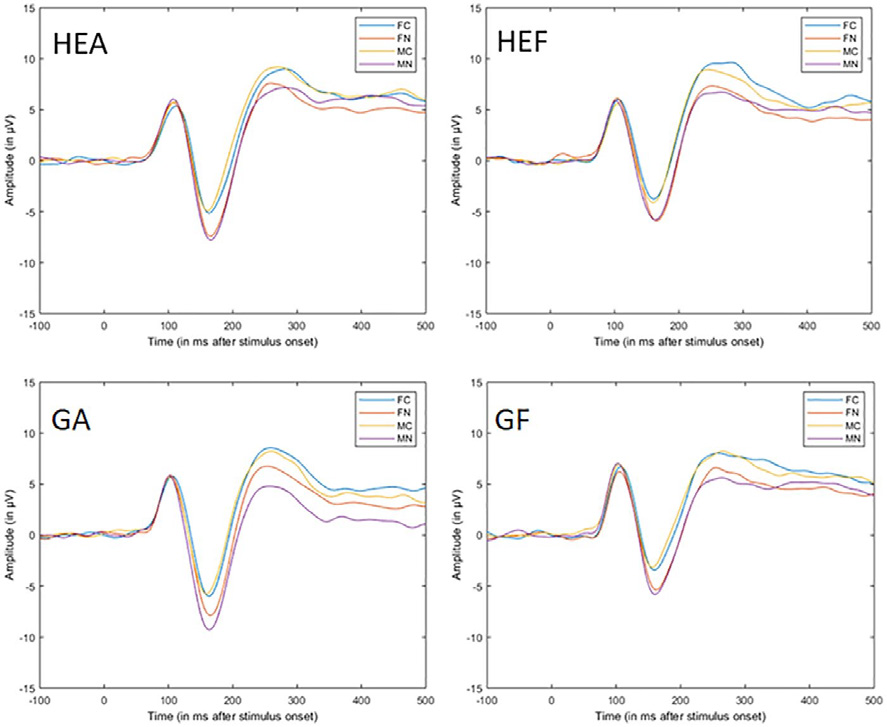
Average ERP across stimulus and groups in the choice reaction time task. *Note.* Average ERP (Pz, O1, O2, P3, and P4) during picture presentation in the choice reaction time task. Stimulus onset is at 0 ms (FC = clothed female, FN = naked female, MC = clothed male, MN = naked male, GA = truthful gay group, GF = lying gay group, HEA = truthful heterosexual group, and HEF = lying heterosexual group). ERP = event-related potential.

**Figure 4.**
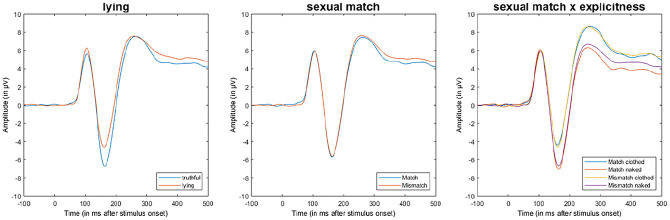
Average ERP across conditions in the choice reaction time task. *Note.* Average ERP (Pz, O1, O2, P3, and P4) for the choice reaction time task showing the effects of the factor lying (left), sexual match (middle), and the interaction of lying and sexual match (right). Stimulus onset is at 0 ms. ERP = event-related potential.

Figure 3 shows the ERP data from the choice reaction time task for the four groups. As expected, explicitness showed the main effect, *F*(1, 75) = 252.71, *p* < .001, η_p_^2^ = 0.77, with naked pictures generally eliciting more pronounced negative EPN. Contrary to expectations, there was no effect of sexual match, *F*(1, 75) = 0.86, *p* = .579, η_p_^2^ < 0.01, and the lying factor, *F*(1, 75) = 3.78, *p* = .055, η_p_^2^ = 0.05 (Figure 4). The expected interaction between explicitness and sexual match showed no significance, *F*(1, 75) = 3.45, *p* = .067, η_p_^2^ = 0.04. Thus, it was not apparent in the EPN when the picture’s gender matched the sexual preference. Although an effect of the naked pictures was observed, this effect was not influenced by pictures matching sexual gender preference. None of the hypotheses were supported by the EPN data. There was an unexpected second-order interaction between explicitness, sexual match, and sexual orientation, *F*(1, 75) = 15.29, *p* < .001, η_p_^2^ = 0.17, as well as a third-order interaction among all factors, *F*(1, 75) = 5.30, *p* = .024, η_p_^2^ = 0.07. There were no other significant effects.

#### Positive slow wave

As expected, explicitness *F*(1, 75) = 81.29, *p* < .001, η_p_^2^ = 0.52, and sexual match showed a main effect, *F*(1, 75) = 9.92, *p* = .002, η_p_^2^ = 0.12, and there was an interaction between the two factors, *F*(1, 75) = 7.8, *p* = .006, η_p_^2^ = 0.09 (Figure 4). There was no significant effect of lying. These results are partly in line with Hypotheses 1 and 2. When the gender of the pictures matched the sexual gender preference of the participants, it resulted in an effect on the PSW but contrary to expectations in terms of a *less pronounced* (positive) PSW. When the gender on the pictures matched sexual preference, explicitness had a larger effect on the PSW compared to when pictures did not match gender preference. There was support for Hypothesis 3 because the lying and truthful groups did not differ at this processing stage.

There were further unexpected interaction effects between sexual orientation and sexual match, *F*(1, 75) = 3.98, *p* = .049, η_p_^2^ = 0.05, and between lying and sexual match, *F*(1, 75) = 10.53, *p* = .001, η_p_^2^ = 0.12, as well as a third-order interaction among all factors, *F*(1, 75) = 5.03, *p* = .027, η_p_^2^ < 0.06.

### Dot Probe Task

#### Accuracy

There were no significant main effects on the accuracy of data: sexual match *F*(1, 74) = 3.54, *p* = .064, η_p_^2^ = 0.05; sexual explicitness *F*(1, 74) = 1.48, *p* = .227, η_p_^2^ = 0.02; lying *F*(1, 74) = 0.38, *p* = .538, η_p_^2^ < 0.01. There was no interaction between sexual match and lying, *F*(1, 74) < 0.01, *p* = .970, η_p_^2^ < 0.01, or between sexual match and explicitness, *F*(1, 74) = 1.55, *p* = .217, η_p_^2^ = 0.02. Again, this supports the notion that participants displayed an equal amount of correct responses throughout all experimental conditions of the dot-probe task.

#### Reaction times

**Figure 5.**
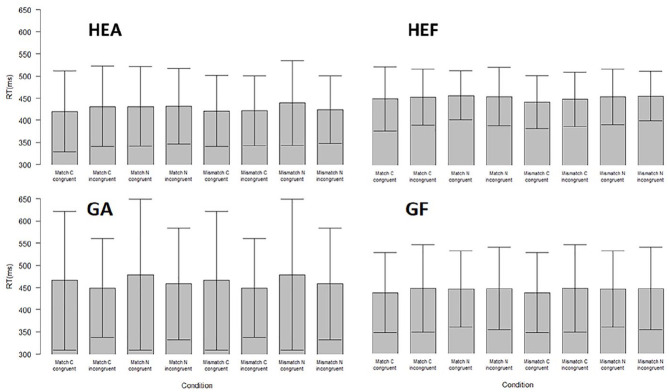
Mean reaction times from the dot-probe task. *Note*. Mean reaction times (RT) and *SD* for the four groups and the different picture conditions in the dot-probe task (GA = truthful gay group, GF = lying gay group, HEA = truthful heterosexual group, and HEF = lying heterosexual group). RT = reaction times; SD = standard deviation.

Figure 5 shows the reaction time results from the dot-probe task. There was no effect of lying and no effect of sexual match but only the main effect of explicitness, *F*(1, 74) = 17.49, *p* < .001, η_p_^2^ = 0.19, with naked pictures resulting in higher mean reaction times. The results do not support Hypotheses 1 and 2 because the sexual match did not influence reaction times. The truthful and lying groups also did not differ in reaction times. This is in support of Hypothesis 3. The effect of explicitness did not interact with congruency, *F*(1, 74) = 2.36, *p* = .128, η_p_^2^ = 0.03. The delayed response for naked pictures was more pronounced in the congruent condition where the dot appeared after the relevant picture instead of the scrambled picture. There was no interaction between congruency and sexual match, *F*(1, 74) = 2.55, *p* = .114, η_p_^2^ = 0.03. When the dot appeared subsequent to the relevant picture content, there was a delayed response for the stimuli not matching sexual interest. When the dot appeared behind the scrambled picture, there was a delayed response for the stimuli matching sexual interest. There was also a second-order interaction between lying, sexual match, and congruency, *F*(1, 74) = 5.05, *p* = .027, η_p_^2^ = 0.06. There were no other significant effects.

**Figure 6.**
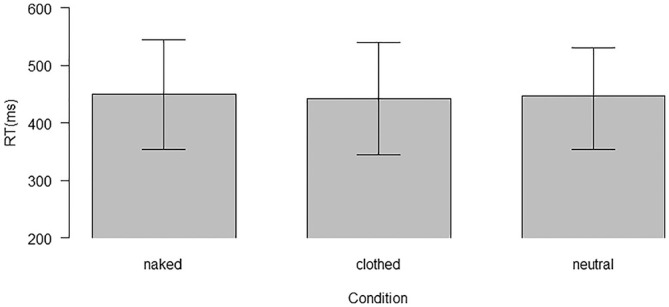
Mean reaction times for naked, clothed, and neutral pictures in the dot-probe task. *Note.* Mean reaction times (RT) for the three trial types (naked, clothed, and neutral) in the dot-probe task. RT = reaction times.

As only explicitness showed a main effect, we used the 80 baseline trials with scrambled pictures followed by a dot to further quantify this effect with exploratory analyses. The reaction times of three trial types (neutral, clothed, naked; see Figure 6) were subjected to a one-way ANOVA. There was a main effect of trial type, *F*(2, 154) = 4.10, *p* = .018, η_p_^2^ = 0.05. However, only the naked and the clothed trials could be differentiated in Bonferroni post hoc pairwise comparisons (naked vs. clothed *p* < .001, naked vs. neutral *p* = .672, clothed vs. neutral *p* = .578). On average, naked trials had longer reaction times (450.05 ms) than the clothed trials (442.53 ms).

To determine whether vigilance or difficulty in disengaging attention was the guiding process behind the delays, additional exploratory analyses were carried out. The neutral, congruent, and incongruent trials were compared separately within the clothed and the naked conditions. Difficulty in disengaging from the picture content would result in a delayed response to incongruent trials compared to neutral trials. Vigilance would be reflected in quicker responses to congruent trials compared to neutral trials. There was, however, no effect of trial type either in the clothed condition, *F*(2, 154) = 1.55, *p* = .213, η_p_^2^ = 0.02, or in the naked condition, *F*(2, 154) = 1.10, *p* = .333, η_p_^2^ = 0.01. Based on these results, no conclusive inferences could be made about whether sexual explicitness influences reaction times through mechanisms of vigilance or difficulty in disengaging.

#### Early posterior negativity

**Figure 7.**
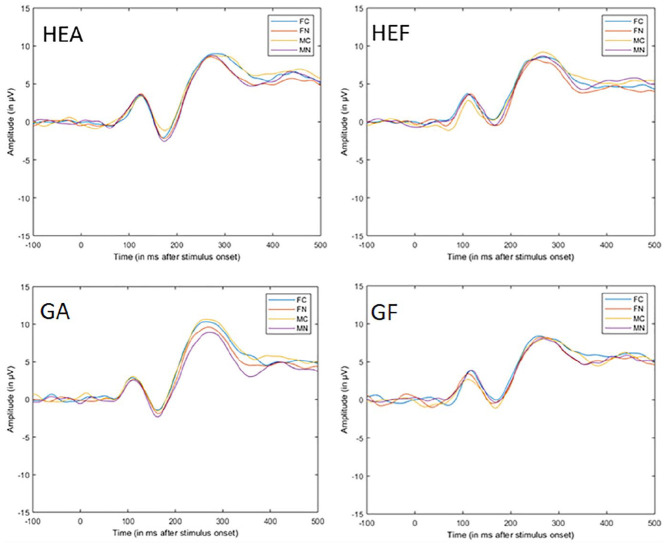
Average ERP across stimulus and groups in the dot-probe task. *Note.* Average ERP (Pz, O1, O2, P3, P4) during picture presentation in the dot-probe task. Stimulus onset is at 0 ms (FC = clothed female, FN = naked female, MC = clothed male, MN = naked male, GA = truthful gay group, GF = lying gay group, HEA = truthful heterosexual group, and HEF = lying heterosexual group). ERP = event-related potential.

Figure 7 shows the ERP data from the dot-probe task for the four groups. As expected, explicitness showed the main effect, *F*(1, 74) = 21.66, *p* < .001, η_p_^2^ = 0.23, with naked pictures eliciting more pronounced negative EPN. Contrary to expectations, there was no effect of sexual match or interaction of sexual match and explicitness and no significant effect of the lying factor, *F*(1, 74) = 3.02, *p* = .086, η_p_^2^ = 0.04, with more pronounced negative EPN in the truthful group. These results did not support Hypotheses 1 and 2 because only the naked pictures influenced the EPN, irrespective of a sexual match. There was also little support for Hypothesis 3, stating no difference between the truthful and lying groups. There was also a third-order interaction among all factors, *F*(1, 74) = 8.05, *p* = .005, η_p_^2^ = 0.10. There were no other significant effects.

#### Positive slow wave

**Figure 8.**
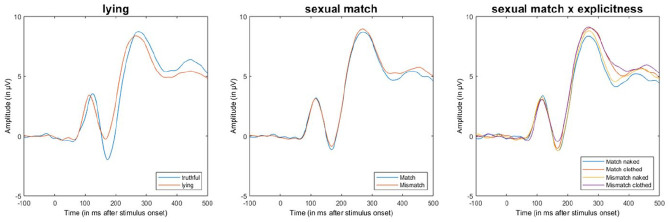
Average ERP across conditions in the dot-probe task. *Note.* Average ERP (Pz, O1, O2, P3, and P4) for the dot-probe task showing the effects of the factor lying (left), sexual match (middle), and the interaction of lying and sexual match (right). Stimulus onset is at 0 ms. ERP = event-related potential.

As expected, explicitness showed the main effect, *F*(1, 74) = 35.75, *p* < .001, η_p_^2^ = 0.33 (more pronounced positive PSW for naked pictures). There was no significant effect of the sexual match factor, *F*(1, 74) = 3.77, *p* = .055, η_p_^2^ = 0.05, and no significant interaction between match and explicitness, *F*(1, 74) = 2.8, *p* = .098, η_p_^2^ = 0.04 (Figure 8). Lying had no significant effect on the PSW. These results were partly in line with Hypotheses 1 and 2. When the gender of the pictures matched the sexual preference of the participants, it resulted in an effect on the PSW, but contrary to expectations, a less pronounced (positive) PSW was observed. When the gender on the pictures matched sexual preference, explicitness had a larger effect on the PSW compared to when pictures did not match gender preference. There was support for Hypothesis 3 because the lying and truthful groups did not differ at this processing stage. There was also a third-order interaction among all factors, *F*(1, 74) = 5.33, *p* = .023, η_p_^2^ = 0.07. There were no other significant effects.

## Discussion

The present study was designed to assess brain responses (ERPs) toward images that did or did not fit the sexual orientation of hetero- and gay male participants. In support of Hypothesis 1, sexual match led to longer reaction times and affected the PSW in the choice reaction time task. Similarly, the explicitness of the stimuli affected these responses. Furthermore, explicitness influenced the reaction times and PSW more when the picture’s gender matched the sexual preference, supporting Hypothesis 2. Most of these effects were absent from the data of the dot-probe task and the EPN data from the choice reaction time task, providing no support for either hypothesis. All measures except the reaction times in the choice reaction task supported Hypothesis 3 in that there was no difference between the lying and truthful groups. No conclusive results on the mechanisms of vigilance or difficulty to disengage could be obtained from exploratory analysis in the dot-probe task.

To gauge the potential automaticity of ERPs regardless of instruction, participants were asked to either act according to or against their individual sexual orientation. This was implemented in two different response tasks that previously showed promising differentiation of hetero- and gay participants and allowed inferences about the cognitive mechanisms at play, the choice reaction time task, and the dot-probe task. It should be mentioned that normal variants of sexual orientation were used because these are easily available in the population at large, with the proportion of gay males estimated at 3.9% (Newport, 2018).^3^ In the long run, understanding potentially automatic processes of stimulus selection could be useful for the assessment of paraphilic variants of human sexuality (e.g., sexual preference for children, i.e., pedophilia).

The visual sexual stimuli used in the study were chosen in such a way that emotional valence and arousal were controlled for. Physical image properties, often neglected as potential confounders, were also controlled. Consequently, differences contingent upon stimulus category should be attributable to sexual attractiveness rather than any other stimulus properties. In addition, comparability of participants’ subgroups was ensured, for instance, in terms of sexual desire or inhibition.

The results showed that explicitness was a driving factor influencing visual attention at all stages of processing. Overall, the choice reaction time task was more suitable for differentiating the conditions of interest in this study with the behavioral data. Behavioral data from the dot-probe task were not influenced by other factors of interest apart from explicitness. Looking at the earliest ERP components in both the choice reaction time and the dot-probe tasks, there was no significant effect for either lying or the interaction of explicitness and sexual match. At the early processing stage of the EPN, however, the encoding of the level of explicitness shown on the screen could be the primary cognitive endeavor. Only at the PSW stage did the sexual match between observed gender and sexual orientation influence the ERP data during the choice reaction time task (not in the dot-probe task) regardless of the instructions to lie. Therefore, the effect of the sexual match was only visible at a later stage compared to explicitness, while explicitness had a larger impact on ERP data even at this later stage.

The results reflected the sequential process of sexual responses (e.g., Spiering et al., 2004). Naked or explicit sexual stimuli seemed to be processed in a quick and unconscious manner through implicit pathways and resulted in physiological activation (EPN). At a later stage (PSW), initial attentional mechanisms allowed for the evaluation of a match between sexual stimulus and sexual orientation, leading to sexual appraisal and the initiation of regulatory mechanisms. This regulation was reflected in the effect of lying observed at the reaction time stage after the PSW. Cognitive science and computational models can be used to study processes of mate choice in healthy humans (Miller & Todd, 1998), and future studies should aim to use ERP data to model sequential stages of neural processing (e.g., Taylor et al., 2019) and see how this can predict responses to sexual stimuli.

Neither sexual match nor lying influenced reaction times in the dot-probe task. Contrary to expectations, in the choice reaction time task, lying influenced reaction times. While the assumed delay from pictures matching the participants’ preference was replicated, even when asked to press a button as quickly as possible, it seemed that purposefully influencing responses within a reasonable time range was also possible. Researchers who intend to bypass self-reports on sexual interest by using reaction time measures should keep this in mind. The PSW data in the choice reaction time task was the only measure in this study where sexual match influenced the data despite the instruction to fake (i.e., no effect of lying was observed in this condition).

One study that investigated deceptive ratings on attractiveness with ERP also found longer reaction times for the deceptive responses, while more positive ERP mean amplitudes were linked to deceptive responses as well (Dong et al., 2010). Although the present study only replicated the finding of longer reaction times, there were similar patterns with the main effect of lying affecting EPN, whereas this early component seemed unaffected by sexual match of the pictures. In the present study, pictures of the entire body were used as stimuli. The study from Dong et al. (2010) focused on attractive and unattractive faces. Face-specific processes might be moderated differently by attempts at deception. Therefore, future studies should investigate whether deceptive judgments on sexual pictures are represented differently in ERP than on neutral stimuli.

### Limitations

Some limitations of this study need to be considered. First, although this was a highly controlled laboratory study, the results remain suggestive until replicated, especially because the effect sizes found were mostly moderate and thus smaller than expected in the power analysis. In addition, results from ERP studies based on amplitude measures are still difficult to interpret (e.g., Ulrich, 2013). While eye-tracking measures are more straightforward in this regard, it is difficult to ascribe detailed meaning beyond statistical evaluations when explaining ERP findings. More control over the perceived picture content could be achieved if the method used to select pictures above would be applied at the subject level. With sufficient trials in a rating task, picture sets could be adjusted according to personal preferences to obtain the maximal sexual relevance for individual participants.

Another limitation of this study is the lack of control over the mental states of lying. The corresponding instructions were understood apparently, and participants familiarized themselves with the task at hand by filling out questionnaires according to the target orientation. Nevertheless, there was no intrinsic motivation to uphold this state throughout the study. Oral reminders were used between the tasks, but subjects could have discontinued lying to reduce mental effort. Experimental motivations to persist with the lying position could be beneficial to ensure compliance with task instructions.

A more obvious issue lies in the way questionnaires were filled out in this study. The researcher was present at all times and even touching the subjects’ head (placing EEG electrodes) while they filled out questionnaires involving highly sensitive and private topics. This allowed for efficient use of the subjects’ time during electrode placement (about 45 minutes), and the researcher was able to quickly respond to any questions or issues of the subjects. At the same time, the researcher’s presence could have influenced the validity of the questionnaire responses. Even with comparable group characteristics based on the questionnaire scores, it remains unclear whether and how groups or individuals were influenced by the researcher’s proximity in their responses on the questionnaires.

Another issue is the lack of transparency in terms of the expected effects on reaction times for the subjects. While lying, subjects could guess what someone with an opposite sexual orientation would be answering in a rating task, they most likely were not aware of how to properly display the expected effects in a performance task measuring reaction times. Although faking good in a cognitive test (displaying higher cognitive performance than actually present) would be problematic, they could always rely on faking bad for the stimuli actually matching their sexual interest. Future studies should consider explaining experimental hypotheses, based on the literature, to subjects participating in the lying condition. This will help them understand what pattern is expected in a truthful condition and also give them an advantage for successful lying within the experimental context. More difficulties become apparent when the findings need to be transferred to benefit a real-life setting.

Hypothetically, an ERP-based test capable of differentiating paraphilic sexual interest based on preconscious measures in a laboratory setting would result in further problems, apart from the development of agreed standards on how to preprocess and analyze the EEG data (e.g., Klawohn et al., 2020). The presence of paraphilic sexual interest has predictive validity for reoffending after a charge or sentence for sexual misconduct (Hanson & Bussière, 1998). Therefore, in this context, it would be useful to have reliable and humane measurements for such disorders. The currently available diagnostic tools have only limited utility (Kingston et al., 2007) and improvements are still being discussed (Seto et al., 2016). ERPs might be a more convenient approach than phallometric measures. Even with an assumed high enough clinical precision for single-subject evaluation, such an ERP-based test would suffer from high context sensitivity (e.g., Li et al., 2019). ERPs measured during voluntary participation in a laboratory will most likely differ from the circumstances under which ERPs are recorded during criminal proceedings with legal consequences at stake.

The form of lying operationalized in the present study does not appropriately model real-life settings. More physiological distress must be assumed in real-life settings. This stress could be indicative of lying but also of the fear of not being perceived as truthful (Suchotzki et al., 2017). In the present study, lying was operationalized as assumed cognitive costs that could influence reaction times. Studies have shown that cognitive load itself can selectively influence reaction times depending on the kind of moral judgment (utilitarian or non-utilitarian) demanded (e.g., Greene et al., 2008). There are far more dynamics at play in a real-life setting that were not appropriately modeled in the present study and require further research. This also applies to the transfer from normal variants of sexual orientation (i.e., gay vs. straight) to paraphilic sexual interests. It is difficult for researchers to access samples with paraphilic sexual interests. In this regard, studies rely on healthy study groups with different sexual orientations as models. Appropriate stimuli depicting children, for instance, to elicit the intended effects for the detection of pedophilia, pose another problem.

## Conclusion

This initial proof-of-concept study showed how ERPs can be used in combination with reaction time measures to potentially facilitate the detection of hidden sexual preferences. The findings did not fully explain the key mechanisms still discussed in information processing models of sexual arousal; however, they were gained with high time-resolving methods in a tightly controlled laboratory setting and thus highlighted an important time window (PSW; 250–500 ms) for future studies in this direction. While initial technical and methodological recommendations can be gained for future laboratory studies, the real challenges lie in the fundamental differences between such laboratory settings and real-life situations, where such measures could ultimately be used. ERP signals are also sensitive to such contextual differences.

## Supplemental Material

sj-pdf-1-sax-10.1177_10790632211024241 – Supplemental material for Automaticity of Early Sexual Attention: An Event-Related Potential Study Click here for additional data file. Supplemental material, sj-pdf-1-sax-10.1177_10790632211024241 for Automaticity of Early Sexual Attention: An Event-Related Potential Study by Anastasios Ziogas, Benedikt Habermeyer, Wolfram Kawohl, Elmar Habermeyer and Andreas Mokros in Sexual Abuse: A Journal of Research and Treatment

## Notes

1. To reduce false discovery rate for the ERP data, all analyses were run again without the factor sexual orientation and the same results were found as reported below.

2. One-way ANOVAs with the group as between-group factor, chi-square tests, or independent *t*-tests were calculated.

3. Percentage of U.S. adult males identifying as lesbian, gay, bisexual, or transgender in 2017.

## References

[bibr1-10790632211024241] Alho J. Salminen N. Sams M. Hietanen J. K. Nummenmaa L. (2015). Facilitated early cortical processing of nude human bodies. Biological Psychology, 109, 103–110. 10.1016/j.biopsycho.2015.04.010 25960070

[bibr2-10790632211024241] Bailey K. West R. Mullaney K. M. (2012). Neural correlates of processing negative and sexually arousing pictures. PLOS ONE, 7, Article e45522. 10.1371/journal.pone.0045522 PMC3447763 23029071

[bibr3-10790632211024241] Bell A. J. Sejnowski T. J. (1995). An information-maximization approach to blind separation and blind deconvolution. Neural Computation, 7, 1129–1159. 10.1109/icassp.1995.479719 7584893

[bibr4-10790632211024241] Briggs K. E. Martin F. H. (2009). Affective picture processing and motivational relevance: Arousal and valence effects on ERPs in an oddball task. International Journal of Psychophysiology, 72, 299–306. 10.1016/j.ijpsycho.2009.01.009 19232373

[bibr5-10790632211024241] Costell R. M. Lunde D. T. Kopell B. S. Wittner W. K. (1972). Contingent negative variation as an indicator of sexual object preference. Science, 177, 718–720. 10.1126/science.177.4050.718 5054151

[bibr6-10790632211024241] de Albuquerque C . (2012). Sexual Inhibition and Sexual Excitation Scales–Short Form (SIS/SES–SF): A validation study of the German Version [Unpublished doctoral thesis]. University of Hamburg.

[bibr7-10790632211024241] Deweese M. M. Robinson J. D. Cinciripini P. M. Versace F. (2016). Conditioned cortical reactivity to cues predicting cigarette-related or pleasant images. International Journal of Psychophysiology, 101, 59–68. 10.1016/j.ijpsycho.2016.01.007 26826400 PMC4853025

[bibr8-10790632211024241] Donchin E. Ritter W. McCallum W. C. (1978). Cognitive psychophysiology: Endogenous components of the ERP. In Callaway E. Tueting P. Koslow S. H. (Eds.), Event-related brain potentials in man (pp. 349–411). Academic Press. 10.1016/B978-0-12-155150-6.50019-5

[bibr9-10790632211024241] Dong G. Wu H. Lu Q. (2010). Attempting to hide our real thoughts: Electrophysiological evidence from truthful and deceptive responses during evaluation. Neuroscience Letters, 479, 1–5. 10.1016/j.neulet.2010.05.014 20470861

[bibr10-10790632211024241] Faul F. Erdfelder E. Buchner A. Lang A. G. (2009). Statistical power analyses using G* Power 3.1: Tests for correlation and regression analyses. Behavior Research Methods, 41, 1149–1160. 10.3758/BRM.41.4.1149 19897823

[bibr11-10790632211024241] Feng C. Wang L. Liu C. Zhu X. Dai R. Mai X. Luo Y. J. (2012). The time course of the influence of valence and arousal on the implicit processing of affective pictures. PLOS ONE, 7, Article e29668. 10.1371/journal.pone.0029668 PMC3266261 22295062

[bibr12-10790632211024241] Feng C. Wang L. Wang N. Gu R. Luo Y. J. (2012). The time course of implicit processing of erotic pictures: An event-related potential study. Brain Research, 1489, 48–55. 10.1016/j.brainres.2012.10.019 23078760

[bibr13-10790632211024241] Geer J. H. Bellard H. S. (1996). Sexual content induced delays in unprimed lexical decisions: Gender and context effects. Archives of Sexual Behavior, 25, 379–396. 10.1007/BF02437581 8836471

[bibr14-10790632211024241] Greene J. D. Morelli S. A. Lowenberg K. Nystrom L. E. Cohen J. D. (2008). Cognitive load selectively interferes with utilitarian moral judgment. Cognition, 107, 1144–1154. 10.1016/j.cognition.2007.11.004 18158145 PMC2429958

[bibr15-10790632211024241] Gronau N. Ben-Shakhar G. Cohen A. (2005). Behavioral and physiological measures in the detection of concealed information. Journal of Applied Psychology, 90, 147–158. 10.1037/0021-9010.90.1.147 15641895

[bibr16-10790632211024241] Hanson R. K. Bussière T. M. (1998). Predicting relapse: A meta-analysis of sexual offender recidivism studies. Journal of Consulting and Clinical Psychology, 66, 348–362. 10.1037/0022-006X.66.2.348 9583338

[bibr17-10790632211024241] Harris G. T. Rice M. E. Quinsey V. L. Chaplin T. C. (1996). Viewing time as a measure of sexual interest among child molesters and normal heterosexual men. Behavior and Research Therapy, 34, 389–394. 10.1016/0005-7967(95)00070-4 8871372

[bibr18-10790632211024241] Hietanen J. K. Kirjavainen I. Nummenmaa L. (2014). Additive effects of affective arousal and top-down attention on event-related brain responses to human bodies. Biological Psychology, 103, 167–175. 10.1016/j.biopsycho.2014.09.003 25224182

[bibr19-10790632211024241] Howard R. C. Longmore F. Mason P. (1992). Contingent negative variation as an indicator of sexual object preference was revisited. International Journal of Psychophysiology, 13, 185–188. 10.1016/0167-8760(92)90057-I 1399757

[bibr20-10790632211024241] Howard R. C. Longmore F. J. Mason P. A. Martin J. L. (1994). Contingent negative variation (CNV) and erotic preference in self-declared homosexuals and in child sex offenders. Biological Psychology, 38, 169–181. 10.1016/0301-0511(94)90037-X 7873701

[bibr21-10790632211024241] Imhoff R. Schmidt A. F. Weiß S. Young A. W. Banse R. (2012). Vicarious viewing time: Prolonged response latencies for sexually attractive targets as a function of task-or stimulus-specific processing. Archives of Sexual Behavior, 41, 1389–1401. 10.1007/s10508-011-9879-1 22218785

[bibr22-10790632211024241] Janssen E. Everaerd W. Spiering M. Janssen J. (2000). Automatic processes and the appraisal of sexual stimuli: Toward an information processing model of sexual arousal. Journal of Sex Research, 37, 8–23. 10.1080/00224490009552016

[bibr23-10790632211024241] Janssen E. Vorst H. Finn P. Bancroft J. (2002a). The Sexual Inhibition (SIS) and Sexual Excitation (SES) scales: I. Measuring sexual inhibition and excitation proneness in men. Journal of Sex Research, 39, 114–126. 10.1080/00224490209552130 12476243

[bibr24-10790632211024241] Janssen E. Vorst H. Finn P. Bancroft J. (2002b). The Sexual Inhibition (SIS) and Sexual Excitation (SES) scales: II. Predicting psychophysiological response patterns. Journal of Sex Research, 39, 127–132. 10.1080/00224490209552131 12476244

[bibr25-10790632211024241] Jasper H. H. (1958). The ten-twenty electrode system of the International Federation. Electroencephalography and Clinical Neurophysiology, 10, 380–375. 10590970

[bibr26-10790632211024241] Kagerer S. Wehrum S. Klucken T. Walter B. Vaitl D. Stark R. (2014). Sex attracts: Investigating individual differences in attentional bias to sexual stimuli. PLOS ONE, 9, Article e107795. 10.1371/journal.pone.0107795 PMC4169562 25238545

[bibr27-10790632211024241] Kingston D. A. Firestone P. Moulden H. M. Bradford J. M. (2007). The utility of the diagnosis of pedophilia: A comparison of various classification procedures. Archives of Sexual Behavior, 36, 423–436. 10.1007/s10508-006-9091-x 17186129

[bibr28-10790632211024241] Klawohn J. Meyer A. Weinberg A. Hajcak G. (2020). Methodological choices in event-related potential (ERP) research and their impact on internal consistency reliability and individual differences: An examination of the error-related negativity (ERN) and anxiety. Journal of Abnormal Psychology, 129, 29–37. 10.1037/abn0000458 31868385 PMC6931902

[bibr29-10790632211024241] Klein F. Sepekoff B. Wolf T. J. (1985). Sexual orientation: A multi-variable. Journal of Homosexuality, 11, 35–49. 10.1300/J082v11n01 4056393

[bibr30-10790632211024241] Knott V. Impey D. Fisher D. Delpero E. Fedoroff P. (2016). Pedophilic potential responses to adult erotic stimuli. Brain Research, 1632, 127–140. 10.1016/j.brainres.2015.12.004 26683083

[bibr31-10790632211024241] Koster E. H. W. Crombez G. Verschuere B. De Houwer J. (2004). Selective attention to threat in the dot-probe paradigm: Differentiating vigilance and difficulty in disengaging. Behaviour Research and Therapy, 42, 1183–1192. 10.1016/j.brat.2003.08.001 15350857

[bibr32-10790632211024241] Kuhn W. Koenig J. Donoghue A. Hillecke T. K. Warth M. (2014). Psychometrische Eigenschaften einer deutschsprachigen Kurzversion des Sexual Desire Inventory (SDI-2) [Psychometric Characteristics of the German Short Version of the Sexual Desire Inventory (SDI-2)]. Zeitschrift Fur Sexualforschung, 27, 138–149. 10.1055/s-0034-1366582

[bibr33-10790632211024241] Kuhr B. Schomberg J. Gruber T. Quirin M. (2013). Beyond pleasure and arousal. NeuroReport, 24, 246–250. 10.1097/WNR.0b013e32835f4eba 23426107

[bibr34-10790632211024241] Lawrence M. A. Lawrence M. M. A. (2016). Package “ez” (R Package Version 4.4-0). https://cran.r-project.org/web/packages/ez/index.html

[bibr35-10790632211024241] Ledoux J. E. (1993). Cognition versus emotion, again-this time in the brain: A response to Parrott and Schulkin. Cognition & Emotion, 7, 61–64. 10.1080/02699939308409176

[bibr36-10790632211024241] Legrand L. B. Del Zotto M. Tyrand R. Pegna A. J. (2013). Basic instinct undressed: Early spatiotemporal processing for primary sexual characteristics. PLOS ONE, 8(7), Article e69726. 10.1371/journal.pone.0069726 PMC3716645 23894532

[bibr37-10790632211024241] Li S. Zhu X. Ding R. Ren J. Luo W. (2019). The effect of emotional and self-referential contexts on ERP responses toward surprised faces. Biological Psychology, 146, 107728. 10.1016/j.biopsycho.2019.107728 31306692

[bibr38-10790632211024241] Miller G. F. Todd P. M. (1998). Mate choice turns cognitive. Trends in Cognitive Sciences, 2, 190–198. 10.1016/S1364-6613(98)01169-3 21227154

[bibr39-10790632211024241] Mokros A. Gebhard M. Heinz V. Marschall R. W. Glasgow D. V. Gress C. L. Z. Laws D. R. (2013). Computerized assessment of pedophilic sexual interest through self-report and viewing time: Reliability, validity, and classification accuracy of the affinity program. Sexual Abuse: A Journal of Research and Treatment, 25, 230–258. 10.1177/1079063212454550 22878565

[bibr40-10790632211024241] Moore D. L. Norris F. H. (2005). Empirical investigation of the conflict and flexibility models of bisexuality. Journal of Bisexuality, 5, 5–25. 10.1300/J159v05n01_02

[bibr41-10790632211024241] Newport F . (2018, May 22). In U.S., estimate of LGBT population rises to 4.5%. Gallup: Politics. https://news.gallup.com/poll/234863/estimate-lgbt-population-rises.aspx

[bibr42-10790632211024241] Oldfield R. C. (1971). Assessment and analysis of handedness: The Edinburgh Inventory. Neuropsychologia, 9, 97–113. 10.1016/0028-3932(71)90067-4 5146491

[bibr43-10790632211024241] Oliver T. L. Meana M. Snyder J. S. (2016). Sex differences in concordance rates between auditory event-related potentials and subjective sexual arousal. Psychophysiology, 53, 1272–1281. 10.1111/psyp.12661 27125689

[bibr44-10790632211024241] Ponseti J. Kropp P. Bosinski H. A. (2009). Brain potentials related to human penile erection. International Journal of Impotence Research, 21, 292–300. 10.1038/ijir.2009.31 19587685

[bibr45-10790632211024241] Prause N. Janssen E. Hetrick W. P. (2008). Attention and emotional responses to sexual stimuli and their relationship to sexual desire. Archives of Sexual Behavior, 37, 934–949. 10.1007/s10508-007-9236-6 17943435

[bibr46-10790632211024241] Prause N. Steele V. R. Staley C. Sabatinelli D. (2015). Late positive potential to explicit sexual images associated with the number of sexual intercourse partners. Social Cognitive and Affective Neuroscience, 10, 93–100. 10.1093/scan/nsu024 24526189 PMC4994844

[bibr47-10790632211024241] Prause N. Steele V. R. Staley C. Sabatinelli D. Hajcak G . (2015). Modulation of late positive potentials by sexual images in problem users and controls inconsistent with “porn addiction.” Biological Psychology, 109, 192–199. 10.1016/j.biopsycho.2015.06.005 26095441

[bibr48-10790632211024241] Price T. F. Dieckman L. W. Harmon-Jones E. (2012). Embodying approach motivation: Body posture influences startle eyeblink and event-related potential responses to appetitive stimuli. Biological Psychology, 90, 211–217. 10.1016/j.biopsycho.2012.04.001 22522185

[bibr49-10790632211024241] Qualls L. R. Hartmann K. Paulson J. F. (2018). Broad autism phenotypic traits and the relationship between sexual orientation and sexual behavior. Journal of Autism and Developmental Disorders, 48, 3974–3983. 10.1007/s10803-018-3556-3 29616484

[bibr50-10790632211024241] Rosenzweig S. (1942). The photoscope is an objective device for evaluating sexual interest. Psychosomatic Medicine, 4, 150–157. 10.1097/00006842-194204000-00004

[bibr51-10790632211024241] Santtila P. Mokros A. Viljanen K. Koivisto M. Sandnabba N. K. Zappalà A. Osterheider M. (2009). Assessment of sexual interest using a choice reaction time task and priming: A feasibility study. Legal and Criminological Psychology, 14, 65–82. 10.1348/135532507X267040

[bibr52-10790632211024241] Schupp H. T. Junghöfer M. Weike A. I. Hamm A. O. (2004). Selective processing of briefly presented affective pictures: An ERP analysis. Psychophysiology, 41, 441–449. 10.1111/j.1469-8986.2004.00174.x 15102130

[bibr53-10790632211024241] Sennwald V. Pool E. Brosch T. Delplanque S. Bianchi-Demicheli F. Sander D. (2016). Emotional attention for erotic stimuli: Cognitive and brain mechanisms. Journal of Comparative Neurology, 524, 1668–1675. 10.1002/cne.23859 26179894

[bibr54-10790632211024241] Seto M. C. Fedoroff J. P. Bradford J. M. Knack N. Rodrigues N. C. Curry S. … Ahmed A. G. (2016). Reliability and validity of the DSM-IV-TR and proposed DSM-5 criteria for pedophilia: Implications for the ICD-11 and the next DSM. International Journal of Law and Psychiatry, 49, 98–106. 10.1016/j.ijlp.2016.08.002 27665026

[bibr55-10790632211024241] Sip K. E. Carmel D. Marchant J. L. Li J. Petrovic P. Roepstorff A. … Frith C. D. (2013). When Pinocchio’s nose does not grow: Belief regarding lie-detectability modulates production of deception. Frontiers in Human Neuroscience, 7, Article 16. 10.3389/fnhum.2013.00016 PMC3563087 23382715

[bibr56-10790632211024241] Snowden R. J. Curl C. Jobbins K. Lavington C. Gray N. S. (2016). Automatic direction of spatial attention to male versus female stimuli: A comparison of heterosexual men and women. Archives of Sexual Behavior, 45, 843–853. 10.1007/s10508-015-0678-y 26857378 PMC4820492

[bibr57-10790632211024241] Spector I. P. Carey M. P. Steinberg L. (1996). The sexual desire inventory: Development, factor structure, and evidence of reliability. Journal of Sex and Marital Therapy, 22, 175–190. 10.1080/00926239608414655 8880651

[bibr58-10790632211024241] Spiering M. Everaerd W. (2007). The sexual unconscious. In Janssen E. (Ed.), Kinsey Institute series: The psychophysiology of sex (pp. 166–184). Indiana University Press.

[bibr59-10790632211024241] Spiering M. Everaerd W. Karsdorp P. Both S. Brauer M. (2006). Nonconscious processing of sexual information: A generalization to women. Journal of Sex Research, 43(3), 268–281. 10.1080/00224490609552325 17599249

[bibr60-10790632211024241] Spiering M. Everaerd W. Laan E. (2004). Conscious processing of sexual information: Mechanisms of appraisal. Archives of Sexual Behavior, 33, 369–380. 10.1023/B:ASEB.0000028890.08687.94 15162083

[bibr61-10790632211024241] Suchotzki K. Verschuere B. Van Bockstaele B. Ben-Shakhar G. Crombez G. (2017). Lying takes time: A meta-analysis on reaction time measures of deception. Psychological Bulletin, 143, 428–453. 10.1037/bul0000087 28182460

[bibr62-10790632211024241] Taylor B. K. Gavin W. J. Grimm K. J. Prince M. A. Lin M. H. Davies P. L. (2019). Towards a unified model of event-related potentials as phases of stimulus-to-response processing. Neuropsychologia, 132, 107128. 10.1016/j.neuropsychologia.2019.107128 31229538 PMC6736637

[bibr63-10790632211024241] Ulrich G. (2013). The theoretical interpretation of electroencephalography: The important role of spontaneous resting EEG and vigilance. BMED Press LLC.

[bibr64-10790632211024241] van Lankveld J. J. D. M. Smulders F. T. Y . (2008). The effect of visual sexual content on event-related potential. Biological Psychology, 79, 200–208. 10.1016/j.biopsycho.2008.04.016 18541359

[bibr65-10790632211024241] Vuilleumier P. (2005). How brain ware: Neural mechanisms of emotional attention. Trends in Cognitive Sciences, 9, 585–594. 10.1016/j.tics.2005.10.011 16289871

[bibr66-10790632211024241] Waismann R. Fenwick P. B. C. Wilson G. D. Hewett T. D. Lumsden J. (2003). EEG responses to visual erotic stimuli in men with normal and paraphilic interests. Archives of Sexual Behavior, 32, 135–144. 10.1023/A:1022448308791 12710828

[bibr67-10790632211024241] Wang Y. F. Jing X. J. Liu F. Li M. L. Long Z. L. Yan J. H. Chen H. F. (2015). Reliable attention network scores and mutually inhibited inter-network relationships revealed by mixed design and non-orthogonal methods. Scientific Reports, 5, 10251. 10.1038/srep10251 25997025 PMC4440527

[bibr68-10790632211024241] Willenbockel V. Sadr J. Fiset D. Horne G. O. Gosselin F. Tanaka J. W. (2010). Controlling low-level image properties: SHINE toolbox. Behavior Research Methods, 42, 671–684. 10.3758/BRM.42.3.671 20805589

[bibr69-10790632211024241] Winkler I. Haufe S. Tangermann M. (2011). Automatic classification of artifactual ICA-components for artifact removal in EEG signals. Behavioral and Brain Functions, 7, 30. 10.1186/1744-9081-7-30 21810266 PMC3175453

[bibr70-10790632211024241] Wright L. W. Adams H. E. (1994). Assessment of sexual preference using a choice reaction time task. Journal of Psychopathology and Behavioral Assessment, 20, 230–231. 10.1007/BF02229209

[bibr71-10790632211024241] Zamansky H. S. (1956). A technique for measuring homosexual tendencies. Journal of Personality, 24, 436–448. 10.1111/j.1467-6494.1956.tb01280.x 13320269

[bibr72-10790632211024241] Ziogas A. Habermeyer E. Santtila P. Poeppl T. Mokros A. (2020). Neuroelectric correlates of human sexuality: A review and meta-analysis. Archives of Sexual Behavior. Advance online publication. 10.1007/s10508-019-01547-3 32016814

