# Supplementary material for: Automaticity of Early Sexual Attention: An Event-Related Potential Study
Source: Sex Abuse. 2021 Jul 8;34(5):507–36. doi: 10.1177/10790632211024241 (PMC9260476; doi:10.1177/10790632211024241)
Supplement: sj-pdf-1-sax-10.1177_10790632211024241 – Supplemental material for Automaticity of Early Sexual Attention: An Event-Related Potential Study [file sj-pdf-1-sax-10.1177_10790632211024241.pdf]

Supplementary Material

Preliminary Study: Selection of Pictures

An additional sample of 40 male participants was recruited for the preliminary study. This sample comprised 20 heterosexual and 20 gay men who did not participate in the main study. The preliminary study was based on self-report data on sexual identity from the Sell Assessment of Sexual Orientation (SASO) in order to assess participants' sexual orientation (Sell, 1996). In the SASO, responses are given on two 7-point Likert-type scales ranging from 1 (*not at all homosexual*) to 7 (*extremely homosexual*) for the homosexual identity scale and from 1 (*not at all heterosexual*) to 7 (*extremely heterosexual*) for the heterosexual identity scale. Values of 4 and above on the heterosexual identity scale are considered indicative of a heterosexual orientation, and scores of 4 and above on the gay identity scale are considered indicative of a gay orientation. Participants also filled out the SDI-2 (Kuhn et al., 2014 based on Spector et al., 1996) and SIS/SES (de Albuquerque, 2012 based on Janssen et al., 2002) questionnaires (see main document for details). Group characteristics from the preliminary study are shown in Supplementary Table 1. A comparison between the sample characteristics from the preliminary study (n = 40) and the main study (n = 80) is presented in Supplementary Table 2.

Supplementary Table 1

Group Characteristics

Variables	HE	Gay	Test Statistic	*df*	*p* Value
N	20	20			
Age (Mean, SD)	26.50 (5.24)	23.25 (3.88)	*t* = 2.23	38	<.05
Age (range)	20-35	18-34			
Sell-HE-ID (Mean, SD)	6.25 (1.07)	1.25 (0.44)	*t* = 19.30	25.36	<.05
Sell-HE-ID (range)	4-7	1-2			
Sell-HO-ID (Mean, SD)	1.1 (0.31)	6.2 (0.77)	*t* = -27.57	24.95	<.05
Sell-HO-ID (range)	1-2	4-7			
SDI-2 Scale 1 (Mean, SD)	25.75 (5.17)	24.35 (4.38)	*t* = 0.31	38	>.05
SDI-2 Scale 2 (Mean, SD)	22.3 (8.49)	25.15 (3.95)	*t* = -1.36	26.86	>.05
SIS 1 (Mean, SD)	8.75 (2.20)	8.3 (1.66)	*t* = 0.73	38	>.05
SIS 2 (Mean, SD)	10.8 (2.82)	11.6 (1.93)	*t* = -1.05	38	>.05
SES (Mean, SD)	16.5 (2.76)	17.3 (2.20)	*t* = -1.01	38	>.05

Note. HE = heterosexual, Sell-HE-ID = Sell heterosexual identity scale (Sell, 1996), Sell-HO-ID = Sell homosexual identity scale (Sell, 1996), SDI-2 Scale 1 = sexual desire with interaction, SDI-2 Scale 2 = sexual desire without interaction (Kuhn et al., 2014 based on Spector et al., 1996), SIS 1 = sexual inhibition due to threat of performance failure, SIS 2 = sexual inhibition due to threat of performance consequences, SES = sexual excitation scale (de Albuquerque, 2012 based on Janssen et al., 2002).

Supplementary Table 2

Comparison between Preliminary Study and Main Study Sample Characteristics

Variables	Prestudy	Main Study	Test Statistic	*df*	*p* Value
N	40	80			
Age (Mean, SD)	24.88 (4.84)	24.73 (4.95)	*t* = 0.16	118	>.05
Age (Range)	18–35	19–43			
SDI-2 Scale 1 (Mean, SD)	25.05 (4.78)	25.76 (4.16)	*t* = -0.85	118	>.05
SDI-2 Scale 2 (Mean, SD)	23.73 (6.70)	24.55 (6.26)	*t* = -0.66	118	>.05
SIS 1 (Mean, SD)	8.53 (1.93)	8.43 (1.76)	*t* = 0.28	118	>.05
SIS 2 (Mean, SD)	11.2 (2.42)	9.8 (2.26)	*t* = 3.07	118	<.01
SES (Mean, SD)	16.9 (2.50)	16.9 (2.39)	*t* = 0.16	118	>.05

Note. SDI-2 Scale 1 = sexual desire with interaction, SDI-2 Scale 2 = sexual desire without interaction (Kuhn et al., 2014 based on Spector et al., 1996), SIS 1 = sexual inhibition due to threat of performance failure, SIS 2 = sexual inhibition due to threat of performance consequences, SES = sexual excitation scale (de Albuquerque, 2012 based on Janssen et al., 2002)

Conducting a preliminary study was necessary to verify the emotional valence ratings for the stimuli used in the main study. Male participants recruited for the preliminary study (*n* = 40) rated all the pictures on three emotional dimensions— valence, arousal, and sexual attractiveness— using a Likert-type scale ranging from 1 to 5 with 1 as the lowest and 5 as the highest rating (programmed with Presentation [Neurobehavioral Systems, Albany, NY, USA]). These ratings were used to ensure that the clothed and naked picture categories mostly differed in sexual attractiveness, whereas, valence and arousal did not differ considerably for these two types of pictures.

Initial analysis showed no rating differences between categories of clothing within each gender category that could be attributed solely to different ratings in the dimension of sexual attractiveness by the appropriate groups (comparisons of clothed females vs. naked females as rated by heterosexuals and clothed male vs. naked male as rated by gay men). As preliminary analyses did not yield the required results, the next step was to select a subset of pictures within each of the four categories so that the new subset would differ between the clothed and naked pictures within a gender category (clothed female to naked female and clothed male to naked male) mostly in terms of sexual attractiveness. For this purpose, the two ratings that should not have differed between clothing conditions (i.e., valence and arousal) were averaged for each picture across all participants from a sexual orientation group. Within each category, this resulted in a list with all pictures and two mean ratings for valence and arousal from one sexual orientation group.

To ensure homogenous ratings in the valence and arousal dimensions across different clothing conditions within a gender category (e.g., between clothed female and naked female), a small range around the median from the average valence and arousal ratings was defined (- 0.1 and + 0.1 around the median on the Likert-type scales for valence and arousal). Only pictures with ratings within those limits on both scales were selected for further

analysis. The goal was to select a very small range within which a high number of pictures would be defined. This resulted in a reduced number of pictures with nearly equivalent ratings in valence and arousal. To ensure differences in sexual attractiveness within this reduced picture set of, say, clothed female pictures, 75% of the pictures with the lowest ratings in sexual attractiveness were selected and within the new reduced set of naked female pictures, 75% of the highest rated pictures in sexual attractiveness were selected. For these new further reduced pictures, the ratings from the heterosexual participants averaged across all pictures within a picture set were used to perform a MANOVA with clothing of picture category (e.g., clothed female and naked female) as the independent variable and the three rating dimensions (valence, arousal, and sexual attractiveness) as dependent measures. The same kind of analysis was performed with 70% of the lowest and highest ratings in sexual attractiveness, with 65% and so on until 5% or only less than three pictures remained in a single picture category. Once these conditions were met, the entire process was repeated but with a larger range around the median (- 0.2 and + 0.2). This was performed automatically until every MANOVA was calculated for all possible constellations within the maximum possible ranges around the medians. As this was not a hypothesis-driven procedure but an explorative search for the best suitable picture set, statistical p values from the MANOVA were used in an iterative fashion to choose the optimal sets. The picture sets were chosen by selecting the significant MANOVAs ($p < .05$) with the highest number of pictures within a set. These picture sets were subjected to follow-up discriminant analyses with ratings as the dependent variable. The same steps were followed for the clothed male and naked male pictures using the ratings by gay participants. Furthermore, an approximately equal number of pictures were chosen for each picture set (clothed female, naked female, clothed male, or naked male).

This procedure led to the selection of 60 clothed females, 60 naked females, 60 clothed males, and 60 naked males. For the heterosexual groups clothed female and naked female pictures represented the picture categories matching sexual orientation, while for the gay groups, the clothed male and naked male pictures represented the picture categories matching sexual orientation in the main study. By combining the ratings of all the 20 heterosexual and 20 gay participants on the selected pictures of the gender matching sexual preference, a MANOVA with picture clothing as the independent variable and the three ratings as the dependent variable was calculated. Using Pillai's trace, a significant effect of clothing ($V = 0.33$, $F[3, 76] = 12.4$, $p < .001$) was found. The follow-up discriminant analysis revealed one discriminant function with coefficients differentiating clothing of picture sets by valence [$b = -0.418$], arousal [$b = -0.226$], and sexual ratings [$b = 1.857$]). Ultimately, the picture sets chosen for each gender differed in sexual attractiveness but not in valence or arousal as rated by the heterosexual and gay participants. The results of the ratings are depicted in Supplementary Figure 1.

Supplementary Figure 1.

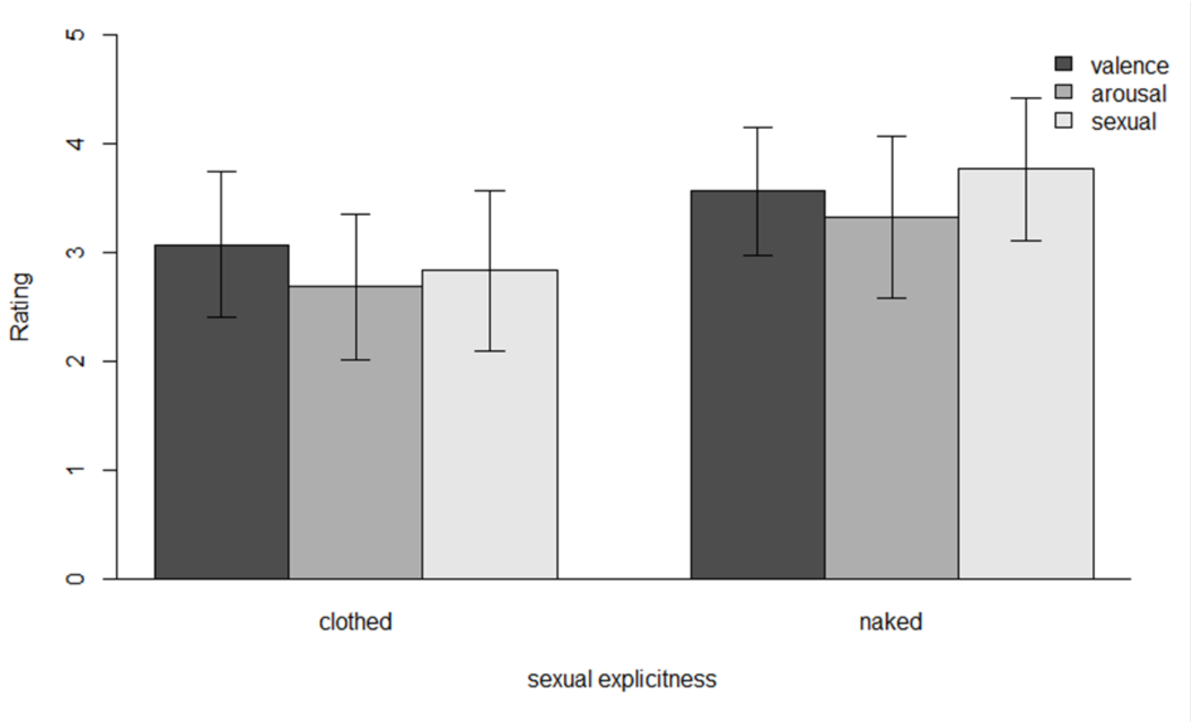

Note. Results from the rating task in the preliminary study mean and SD for valence, arousal, and sexual attractiveness ratings are depicted for the clothed picture categories and the naked picture categories.

The picture set chosen was rated again in the main study by a part of the main sample. Only a subset of participants within the lying groups (10 heterosexual participants and 11 gay participants) also gave untrue responses in the main study rating task as well. This resulted in 59 truthful ratings within the main study to replicate the controlled emotional dimensions. The three ratings for the clothed (sexually non-explicit) and naked (sexually explicit) pictures (independent variable) of the picture category matching the participants' sexual orientation were used as the dependent variable in a MANOVA. Using Pillai's trace, a significant effect of sexual explicitness for the picture category matching the participants' sexual orientation ($V = 0.25$, $F[3, 114] = 12.13$, $p < .001$) was found. A follow-up discriminant analysis revealed one discriminant function with coefficients differentiating sexual explicitness of picture sets by valence [$b = -0.715$], arousal [$b = 0.485$], and sexual attractiveness ratings [$b = 1.531$]). Supplementary Figure 2 displays the ratings (main study) for the two picture sets.

Supplementary Figure 2

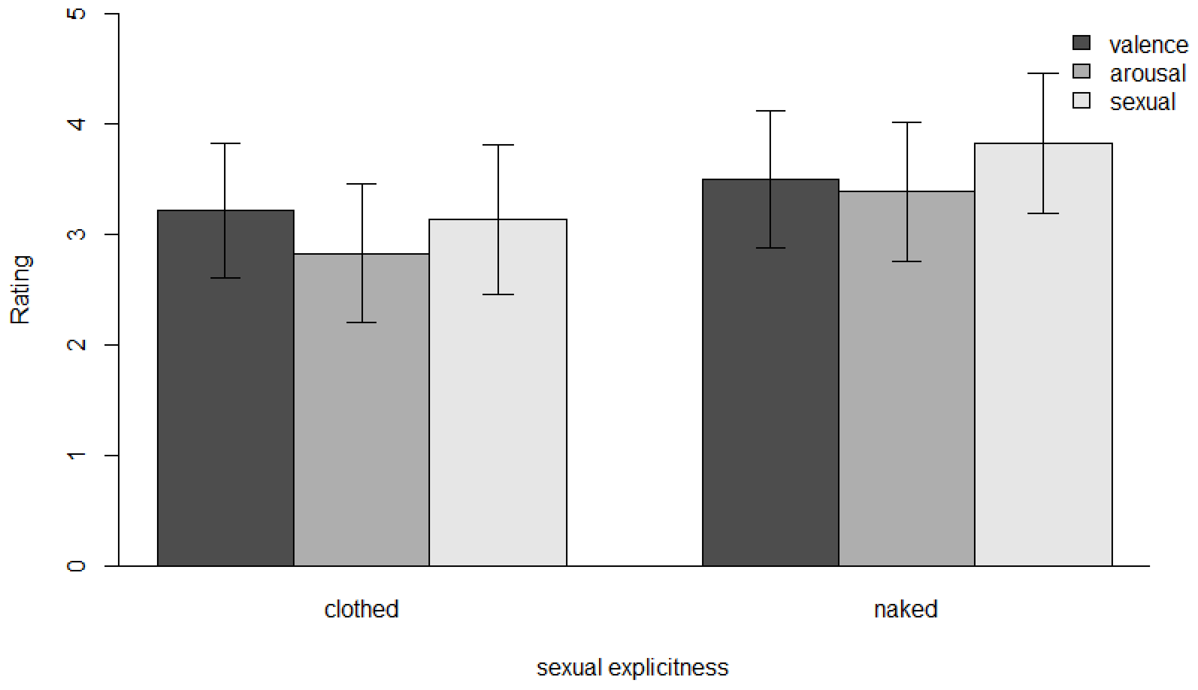

Note. Results from the rating task in the main study, mean and SD for valence, arousal, and sexual attractiveness ratings are depicted for the clothed picture and the naked picture categories.

Truthful and Untrue Responses on the Klein Sexual Orientation Grid

To check if the instructions on lying were understood and executed, KSOG data within the lying conditions were compared. Both groups that were asked to give false responses in the KSOG also gave additional truthful responses. The results are depicted in Supplementary Figure 3. For the two groups within the lying condition, an overall mixed-design ANOVA with the factors of sexual orientation (2: heterosexual vs. gay) and lying (2: lying vs. truthful) was calculated for the KSOG scores. There was no effect of sexual orientation, $F(1, 38) = 2.78$, $p = .103$, $\eta_p^2 = 0.07$, or lying, $F(1, 38) < 0.01$, $p = .999$, $\eta_p^2 < 0.01$. There was, however, an interaction effect between the two factors, $F(1, 38) = 1100.00$, $p < .001$, $\eta_p^2 = 0.97$. These results show that participants followed the instructions to lie adequately.

Supplementary Figure 3

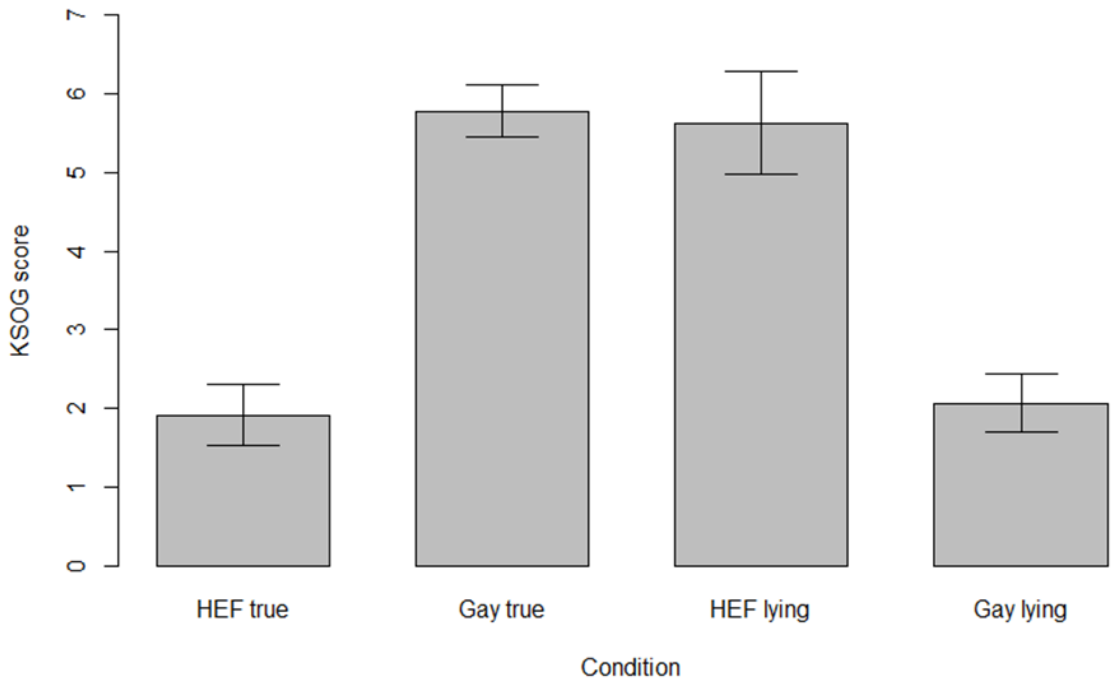

Note. Mean KSOG scores and SD for the two groups (HEF true = lying heterosexual group giving truthful KSOG responses, Gay true = lying gay group giving truthful KSOG responses, HEF lying = lying heterosexual group giving false KSOG responses, Gay lying = lying gay group giving false KSOG responses).

Trial Composition Within the Dot Probe Task

Supplementary Table 3 gives an overview of the different trials used in the dot-probe task (the colors refer to the picture categories).

Supplementary Table 3

Different Trial Types Within the Dot Probe Task and the Number of Trials

Picture left	Picture right	Dot left	Dot right	No dot	Total no. of trials
Neutral	FC	20	20	20	60
Neutral	FN	20	20	20	60
Neutral	MC	20	20	20	60
Neutral	MN	20	20	20	60
FC	Neutral	20	20	20	60
FN	Neutral	20	20	20	60
MC	Neutral	20	20	20	60
MN	Neutral	20	20	20	60
Neutral	Neutral	40	40	40	120
		200	200	200	600

Note. FC, female clothed; FN, female naked; MC, male clothed; MN, male naked.

References

de Albuquerque, C. (2012). The Sexual Inhibition and Sexual Excitation Scales–Short Form (SIS/SES–SF): A validation study of the German Version. *Unpublished Doctoral Thesis, University of Hamburg, Hamburg, Germany.*

Janssen, E., Vorst, H., Finn, P., & Bancroft, J. (2002). The Sexual Inhibition (SIS) and Sexual Excitation (SES) scales: I. Measuring sexual inhibition and excitation proneness in men. *Journal of Sex Research*, 39, 114–126. https://doi.org/10.1080/00224490209552130

Kuhn, W., Koenig, J., Donoghue, A., Hillecke, T. K., & Warth, M. (2014). Psychometrische Eigenschaften einer deutschsprachigen Kurzversion des Sexual Desire Inventory (SDI-2). *Zeitschrift Fur Sexualforschung*, *27*, 138–149. https://doi.org/10.1055/s-0034-1366582

Sell, R. L. (1996). The Sell assessment of sexual orientation: Background and scoring. *Journal of Gay, Lesbian, and Bisexual Identity, 1*, 295–310.

Spector, I. P., Carey, M. P., & Steinberg, L. (1996). The sexual desire inventory: Development, factor structure, and evidence of reliability. *Journal of Sex and Marital Therapy, 22*, 175–190. https://doi.org/10.1080/009262396084146
